# Adjuvant Interventions in Levofloxacin-Exposed Rabbit Achilles Tendons: An Exploratory Controlled Pilot Study with Histopathological and Ultrasonographic Endpoints

**DOI:** 10.3390/jcm15176596

**Published:** 2026-08-26

**Authors:** Oana-Maria Mișcă, Liviu-Coriolan Mișcă, Andreea-Adriana Neamțu, Laura Maghiar, Cristian Constantin Croicu, Flavia Baderca, Amalia Raluca Ceaușu, Oana Cristina Radulescu, Valentin-Cristian Iovin, Titus Grecu, Roxana-Cristina Grecu, Alexandra-Magdalena Ioana, Petrișor Zorin Crăiniceanu, Andrei Gheorghe Marius Motoc

**Affiliations:** 1 Doctoral School Department, “Victor Babeș” University of Medicine and Pharmacy Timișoara, Eftimie Murgu Square No. 2, 300041 Timișoara, Romania; oamarginean@gmail.com (O.-M.M.); oana.radulescu@umft.ro (O.C.R.); valentin.iovin@umft.ro (V.-C.I.); alexandra.ioana@umft.ro (A.-M.I.); 2 Aspire Orthopaedics, UPMC Sports Surgery Clinic, Northwood Avenue, Santry Demesne, D09 C523 Dublin, Ireland; miscal.liviu@gmail.com; 3 Department of Toxicology, Faculty of Pharmacy, “Victor Babeș” University of Medicine and Pharmacy Timișoara, Eftimie Murgu Square No. 2, 300041 Timișoara, Romania; 4 Research Centre for Pharmaco-Toxicological Evaluation, Faculty of Pharmacy, “Victor Babeș” University of Medicine and Pharmacy Timișoara, Eftimie Murgu Square No. 2, 300041 Timișoara, Romania; 5 Department of Pathology, Clinical County Emergency Hospital of Arad, Andrenyi Karoly Str. No. 2–4, 310037 Arad, Romania; 6 Department of Pathology, “Pius Brînzeu” Clinical County Emergency Hospital Timișoara, Liviu Rebreanu Boulevard No. 156, 300723 Timișoara, Romania; 7 Department of Psycho-Neurosciences and Rehabilitation, Faculty of Medicine and Pharmacy, University of Oradea, Universității Str. No. 1, 410087 Oradea, Romania; 8 Department of Dermatovenerology, Clinical County Emergency Hospital Bihor, Gheorghe Doja Str. No. 65, 410169 Oradea, Romania; 9 Veterinaria—Veterinary Orthopedics and Traumatology Center Timișoara, Constantin Brâncoveanu Blvd., No. 125a, 300502 Timișoara, Romania; cristiancroicu@yahoo.com; 10 Department of Microscopic Morphology/Histology, “Victor Babeș” University of Medicine and Pharmacy Timișoara, 300041 Timișoara, Romania; flavia.baderca@umft.ro (F.B.); ra.ceausu@umft.ro (A.R.C.); 11 Angiogenesis Research Centre, “Victor Babeș” University of Medicine and Pharmacy, 300041 Timișoara, Romania; 12 Service of Pathology, Emergency City Hospital, 300254 Timișoara, Romania; 13 Department of Plastic Surgery, The Christie NHS Foundation Trust, 550 Wilmslow Road, Manchester M20 4BX, UK; titus.grecu@nhs.net; 14 Plastic and Reconstructive Surgery Department–Casa Austria, “Pius Brînzeu” Clinical County Emergency Hospital Timișoara, Liviu Rebreanu Boulevard No. 156, 300723 Timișoara, Romania; roxana.totorean@gmail.com (R.-C.G.); zcrainiceanu@gmail.com (P.Z.C.); 15 Department of Anatomy and Embryology, “Victor Babeș” University of Medicine and Pharmacy Timișoara, Eftimie Murgu Square No. 2, 300041 Timișoara, Romania; amotoc@umft.ro

**Keywords:** Achilles tendinopathy, fluoroquinolone, levofloxacin, platelet-rich plasma, injectable collagen, vitamin E, selenium, essential oils, augmented soft-tissue mobilization, Bonar score

## Abstract

**Background/Objectives:** Fluoroquinolones cause Achilles tendinopathy within a narrow, early therapeutic window, yet preventive strategies given alongside these antibiotics remain largely untested. This exploratory pilot study assessed whether four mechanistically distinct adjuvants attenuated histopathological tendon change in a rabbit model of levofloxacin exposure. **Methods:** Twenty-one male New Zealand White rabbits received oral levofloxacin (100 µg/kg/day, seven days). A contralateral-limb design yielded 42 tendons (two cohorts provided paired limb-specific comparisons and a third cohort received the systemic intervention). Animals were allocated to various groups, receiving platelet-rich plasma (PRP), injectable porcine collagen, oral vitamin E + selenium, topical essential oils with augmented soft-tissue mobilization (ASTM), or a levofloxacin-only control. The main outcome was a modified Bonar composite (tenocyte morphology, cellularity, vascularity, paratendinous fibrosis; 0–12) at three months; additional outcomes were the individual domains and ultrasonographic tendon thickness. **Results:** Composite scores were low throughout (group medians 2.0–4.0) and did not differ between groups (H = 3.663, *p* = 0.45); no domain differed, and the untreated control did not show the greatest change. Within animals, collagen-treated tendons scored higher than the paired essential-oil tendons (median difference +3.0, nominal *p* = 0.031, Bonferroni-adjusted *p* = 0.062). Tendon thickness increased by day 14 only with vitamin E + selenium (+14.1%; *p* = 0.003, and *p* = 0.006 adjusted for baseline); this finding arose in a separate cohort with thinner baseline tendons, cannot be confidently attributed to the intervention, and was unrelated to histopathology (ρ = −0.02, *p* = 0.91). Macroscopic findings differed between groups (nominal *p* = 0.031) without predicting tissue grade. **Conclusions:** No statistically detectable difference in the study-specific four-domain composite was identified among the treatment conditions; the study lacked a levofloxacin-free control and could not provide a definitive efficacy assessment. The model produced only mild pathology, limiting its power, and neither imaging nor gross inspection predicted tissue-level change. These preliminary observations are hypothesis-generating and are intended to inform the design of an adequately powered confirmatory study. The findings are inconclusive for efficacy; the design cannot distinguish lack of adjuvant efficacy from failure of the model to induce measurable disease.

## 1. Introduction

Tendinopathy is a chronic, painful, and frequently disabling disorder characterized by load-related pain, impaired function, and localized swelling [1,2]. The Achilles tendon carries one of the greatest burdens. Midportion disease affects an estimated 4–7% of the general population and more than half of elite endurance runners [1,2], with a high incidence also documented in military personnel [3] and recreational runners [4], while Achilles rupture remains the most common tendon rupture worldwide [5], with multicentric epidemiological data implicating dysmetabolic diseases as frequent cofactors of rupture [6]. Progression is most commonly framed by the Cook–Purdam continuum of reactive, disrepair, and degenerative stages [7,8]. Tenocytes act as mechanosensitive regulators of extracellular matrix (ECM) homeostasis, and dysregulated matrix metalloproteinase (MMP) activity drives progression toward irreversible degeneration [9], implying that intervention is most likely to succeed before the degenerative stage is reached [7,8].

Fluoroquinolones (FQs), particularly, ciprofloxacin and levofloxacin, are among the most widely prescribed antibiotics worldwide and are associated with Achilles tendinopathy or rupture in approximately 0.1–0.4% of treated patients [10,11]. Pharmacovigilance analyses have identified strong disproportionality signals for both tendonitis and rupture, with levofloxacin most strongly associated with rupture [12]; nationwide cohort data confirm a positive exposure–outcome association [13], as do comparative analyses against non-FQ regimens for the same indication [14]. These concerns led the European Medicines Agency to restrict systemic FQ prescribing and later to reiterate those restrictions after evidence of only modest change in practice [15].

Effects may include chelation of divalent cations, impaired tenocyte proliferation and migration, and marked oxidative stress with antioxidant depletion and mitochondrial dysfunction in tendon-resident cells [10,16,17]. Onset is early and unpredictable, with most cases arising within 30 days of treatment at a median of 6–9 days, though symptoms may appear months after discontinuation [11,17]. These features define a narrow therapeutic window in which prevention is biologically more rational than treatment introduced after structural damage or rupture [16]. The literature nonetheless remains focused on post-injury management, and comparative data on adjuvants given concomitantly with the causative antibiotic are scarce, as also apparent from systematic reviews of the biological and chemical changes underlying fluoroquinolone-associated tendinopathy, which focus on mechanism and management rather than on concomitant prophylaxis [18].

Among regenerative options, platelet-rich plasma (PRP) is the most extensively studied. On activation, it releases PDGF, TGF-β, VEGF, bFGF, and IGF-1, promoting angiogenesis, fibroblast proliferation and collagen synthesis during early healing [19,20,21]. Clinical evidence in chronic Achilles tendinopathy is nevertheless heterogeneous, with recent meta-analyses reporting inconsistent and frequently non-significant effects on pain and function [22,23,24] and attenuated responses in metabolically compromised patients [25]. Tom our knowledge, whether early prophylactic PRP prevents FQ-induced tendinopathy, rather than treating established disease, has not been investigated. Injectable type I porcine collagen (MD-Tissue^®^, Guna) offers a minimally invasive, scaffold-based alternative; cultured human tenocytes exposed to it show increased COL-I synthesis, maturation and secretion, upregulation of TIMP-1, and enhanced migration through mechanotransduction-related pathways [26,27]. Because it targets two principal FQ-induced alterations, i.e., reduced COL-I production and increased MMP activity, it is biologically plausible as prophylaxis, but it remains untested in this context.

Given the central role of oxidative stress, antioxidant supplementation is a further mechanism-oriented strategy. Vitamin E (α-tocopherol) protects membranes against lipid peroxidation and supports fibroblast proliferation and collagen synthesis [28,29], whereas selenium is an essential cofactor of selenoproteins including the glutathione peroxidases [30], with combined supplementation appearing synergistic [28,31]. A prospective randomized pilot study by our team found that concomitant vitamin E and selenium during levofloxacin therapy was associated with lower VAS pain scores, improved VISA-A scores, and fewer ultrasonographic signs of tendinopathy at three months [16], supporting controlled preclinical evaluation.

A fourth approach combines local anti-inflammatory action with mechanical stimulation. Augmented soft tissue mobilization (ASTM) is a friction-based technique shown to activate fibroblasts and stimulate fibronectin and COL-I production [32], with systematic review evidence of outcomes at least comparable to conventional interventions [33]. Topical formulations containing balsam fir (*Abies balsamea*), lemon eucalyptus (*Eucalyptus citriodora*), katrafay (*Cedrelopsis grevei*), Italian immortelle (*Helichrysum italicum*), and mastic tree (*Pistacia lentiscus*) exploit the anti-inflammatory and analgesic properties of their terpene constituents, with in vitro data confirming inhibition of inflammatory mediators and lipid peroxidation [34] and meta-analyses supporting topical essential-oil analgesia in musculoskeletal disorders [35]. Their combination has nevertheless not previously been tested as prophylaxis against drug-induced tendinopathy.

Preclinical assessment is complicated by the absence of an accepted model. Chemically and mechanically induced models differ in severity, time course and validity, each reproducing only selected aspects of the human disease [36,37,38], and a recent scoping review concluded that promising preclinical results translate poorly, partly because of heterogeneous induction protocols and high risk of bias [39]. Models that combine a structural endpoint with inflammatory or nociceptive readouts in joint-adjacent tissues [40] represent one strategy for increasing the sensitivity of small-animal designs. Oral FQ administration reproduces the clinically relevant exposure route and has been used in rodent and rabbit studies [41,42,43], but the severity of resulting histopathological change varies considerably between protocols. The present regimen (oral levofloxacin 100 µg/kg/day for seven days, with histological assessment at three months) was selected pragmatically; however, in the absence of a validated induction protocol, the severity and persistence of histological change achievable with this regimen could not be guaranteed a priori, and this uncertainty is central to the interpretation of the results.

Against this background, the aim of this exploratory pilot study was to evaluate, in levofloxacin-exposed rabbits, whether four mechanistically distinct adjuvant therapies—local PRP infiltration, local injectable porcine collagen, oral vitamin E and selenium, and topical essential oils delivered through ASTM—modified the development of histopathological tendinopathy when administered concomitantly with the antibiotic and maintained throughout the highest at-risk period. The main outcome was a semi-quantitative modified Bonar score on longitudinal Achilles tendon sections harvested three months after the start of antibiotic exposure. Because no comparative preclinical data existed for any of these adjuvants in the adopted context, the study was designed as an exploratory pilot comparison against a levofloxacin-only control, intended to establish feasibility, estimate the direction and variability of any effect, and inform the design of a subsequent adequately powered trial, rather than as a confirmatory test of a predicted protective effect.

## 2. Materials and Methods

### 2.1. Ethical Approval and Regulatory Compliance

The animal protocol was approved by the Ethics Committee of the Victor Babeș University of Medicine and Pharmacy, Timișoara, Romania (Approval No. 64/17 December 2020), by the Institutional Review Board and Ethics Committee for Scientific Research of the Pius Brînzeu Emergency Clinical County Hospital, Timișoara, Romania (Approval No. 223/5 February 2021), and by the Directorate for Veterinary and Food Safety of Timiș County (Direcția Sanitară Veterinară și pentru Siguranța Alimentelor Timiș, DSVSA Timiș, Romania) under Project Authorization No. 009/24 September 2021. All experimental procedures complied with Government Ordinance No. 42/2004 and with European Directive 2010/63/EU on the protection of animals used for scientific purposes. Reporting follows the ARRIVE 2.0 guidelines. The completed ARRIVE 2.0 checklist is provided in the Supplementary Materials. The experiments were carried out between 24 September 2021 and 24 December 2021.

### 2.2. Study Design and Outcome Measures

This was a prospective, controlled exploratory pilot study. Structurally, it comprised three distinct sub-experiments rather than a conventional five-arm randomized trial: two within-animal paired comparisons (injectable collagen versus essential oils + ASTM, and PRP versus a levofloxacin-only control), in which the tendon was the treated unit and the animal served as its own block, and a separate seven-animal cohort receiving a systemic intervention (vitamin E + selenium), for which the animal was the experimental unit. It was a pilot study using a rabbit model of oral levofloxacin exposure. It was designed to assess feasibility, to estimate the direction and variability of any treatment effect, and to inform the design of a subsequent adequately powered trial, rather than to provide confirmatory evidence of efficacy. The research question, key design features, and analysis plan were specified in the study protocol approved by the ethics committees and a competent veterinary authority (Section 2.1); the protocol was not additionally registered in a public registry. All animals received levofloxacin; they were then allocated to four adjuvant-therapy interventions or to a levofloxacin-only control, with a total follow-up of three months from the first antibiotic dose.

The main outcome was the composite modified Bonar histopathological score (range 0–12) measured on Achilles tendon sections harvested at the end of follow-up. Additional outcomes were the four individual histopathological domain scores and ultrasonographic Achilles tendon thickness, measured in vivo at each examination and ex vivo on the harvested specimen. Additional descriptive data collected for every tendon or animal included the following: (i) macroscopic findings at dissection; (ii) qualitative B-mode ultrasonographic descriptors at three timepoints; and (iii) body weight, food intake and general behavior as tolerability measures. No functional, biomechanical, biochemical, or molecular outcomes were assessed, and no behavioral pain scoring was performed.

### 2.3. Animals, Housing and Husbandry

Twenty-one skeletally mature male New Zealand White rabbits (*Oryctolagus cuniculus*), aged 3–4 years and weighing 3.40–4.74 kg at baseline (mean 4.11 kg), were obtained from the Cantacuzino National Institute of Medical-Military Research and Development (Bucharest, Romania). Each animal was implanted with a subcutaneous microchip carrying a unique alphanumeric code, to ensure unambiguous identification and to prevent allocation errors after randomization. Animals were housed individually at 20 ± 2 °C and 50 ± 10% relative humidity under a 12 h light/dark cycle, with ad libitum access to commercial rabbit chow and water throughout the study. Body weight, food intake, and general behavior were monitored daily by the attending staff in accordance with institutional animal welfare guidelines. Animals were allowed free locomotion in their enclosures between procedures, providing physiological tendon loading. No animal was excluded, and there was no attrition. All 21 rabbits completed the three-month protocol, and all 42 Achilles tendons were available for analysis.

### 2.4. Sample Size, Randomization, Allocation and Blinding

Consistent with an exploratory pilot design, no a priori statistical power calculation was performed and no minimum detectable effect was specified; the sample size was determined pragmatically by the number of animals available under the approved protocol and by the 3Rs principle of reduction, exploiting a contralateral-limb design to obtain 42 analyzable tendons from 21 animals. A post hoc sensitivity analysis based on noncentral F and t distributions indicated that, at a two-sided alpha of 0.05 and 80% power, the five-group comparison of 42 tendons could detect only a very large between-group effect (Cohen’s f of approximately 0.57, corresponding to an eta-squared of approximately 0.24); the achieved power was approximately 19% for a conventional medium effect (f = 0.25) and 47% for a large effect (f = 0.40). For the within-animal comparisons based on seven pairs, the minimum detectable paired effect size at 80% power was a Cohen’s dz of approximately 1.27, and even a conventionally large paired effect (dz = 0.8) would be detected only approximately 43% of the time. These figures are intended to inform the design of a subsequent adequately powered study.

Animals were randomly allocated to the five experimental groups and were identified throughout the study by their individual microchip codes. Allocation was performed by sealed-envelope randomization carried out by the principal investigator. Sealed opaque envelopes, prepared and drawn by the principal investigator, were used for the allocation of animals among the cohorts. The five treatment conditions were nevertheless not randomized independently of cohort; thus, the treatment remained partially confounded with cohort membership.

The histopathologists who scored all slides and the physician who performed the ultrasonographic examinations were blinded to group allocation, and slides were coded so that group identity could not be inferred during assessment. Blinding was not feasible for the personnel who administered the interventions, since the procedures differed visibly between groups.

### 2.5. Levofloxacin Exposure (Intended Induction of Tendinopathy)

Tendinopathy induction was attempted pharmacologically in all 21 animals by oral administration of levofloxacin liquid solution at 100 µg/kg/day for seven consecutive days, beginning on day 1 of the protocol. Levofloxacin was supplied as a sterile solution containing 500 mg levofloxacin (as levofloxacin hemihydrate) in 100 mL, equivalent to 5 mg/mL (Levofloxacin Kabi^®^ 5 mg/mL; Fresenius Kabi, Bad Homburg, Germany), with sodium chloride, hydrochloric acid, sodium hydroxide, and water as excipients. Although the preparation was formulated for intravenous infusion, it was administered orally at the prescribed dose throughout the study. The daily dose was administered orally by syringe as a single daily administration; liquid formulation was preferred because oral dosing of rabbits is more reliable with a liquid than with solid formulations. The pH, excipient composition, and stability data were those of the commercial preparation, and no additional pharmaceutical characterization was performed within this study. Moreover, the administered dose of 100 µg/kg/day was substantially lower than the milligram-per-kilogram doses used in the cited rodent studies, and this low exposure is itself a plausible contributor to the mild histopathological changes observed (Section 4). This dose and schedule were adapted from previously published rodent models of oral fluoroquinolone-induced Achilles tendinopathy [42,43] and from a rabbit study using oral levofloxacin at a comparable dose without overt systemic toxicity [41], scaled to the duration of a standard human levofloxacin course. This scaling was pragmatic rather than pharmacokinetic or allometric; the cited rabbit study employed oral levofloxacin in an infection model and was not designed to establish tendon toxicity, the intravenous formulation used here has not been validated for oral administration in rabbits, and serum levofloxacin concentrations were not measured; so, the systemic and intratendinous exposure actually achieved is unknown.

### 2.6. Experimental Groups and Tendon Allocation

**Figure 1:**
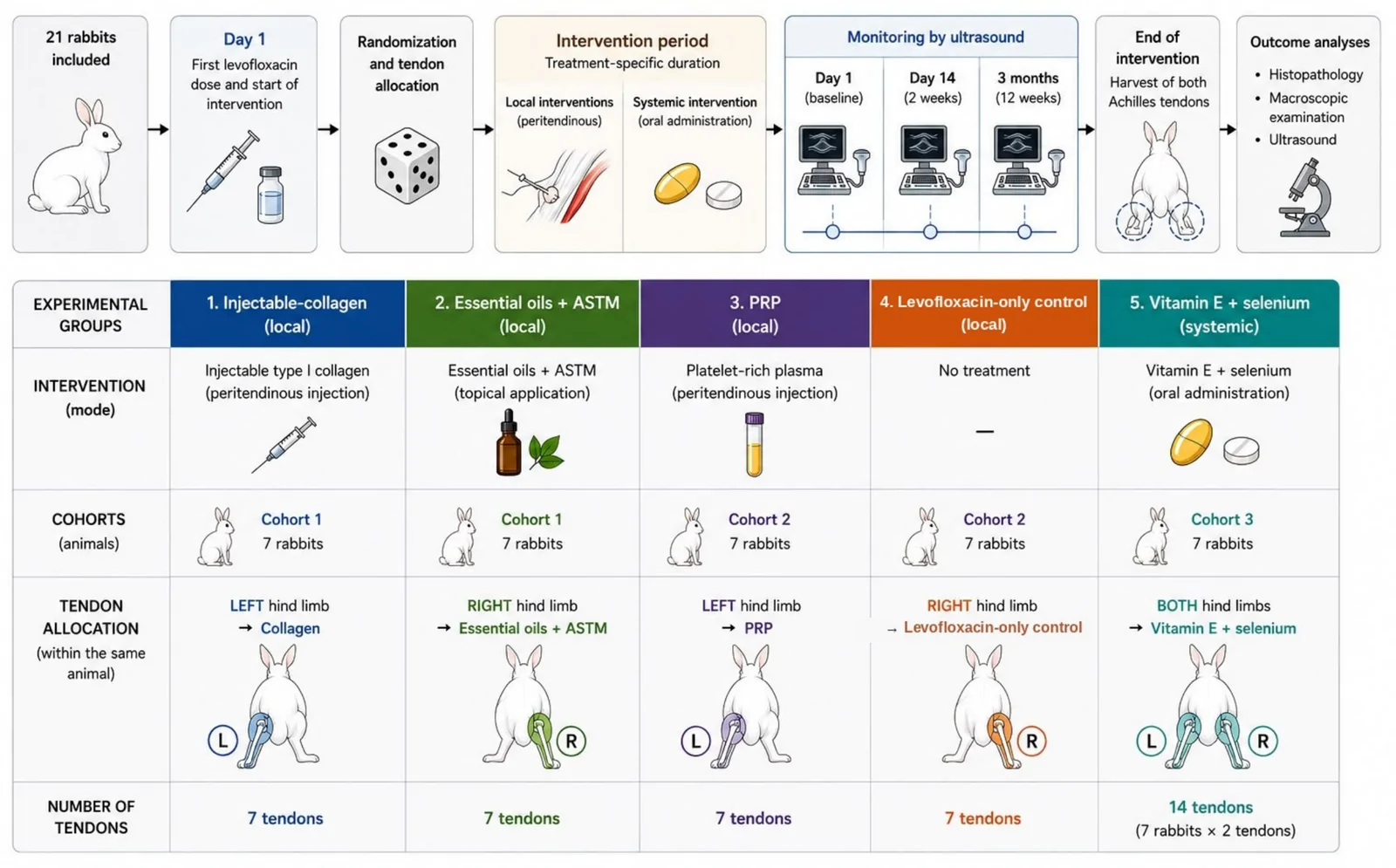
Study flow and experimental design: cohort formation, randomization and tendon allocation, treatment-specific intervention periods, ultrasonographic monitoring schedule, and outcome analyses. Each of the three cohorts of seven animals contributed both hind limbs to the study; in cohorts 1 and 2, the two limbs of the same animal were allocated to different groups, whereas in cohort 3, both limbs received the same systemic intervention. ASTM, augmented soft-tissue mobilization; PRP, platelet-rich plasma.

Animals were allocated to five groups (Figure 1). In the four adjuvant-treatment groups the intervention began on day 1, concomitantly with the first levofloxacin dose, and continued for the treatment-specific duration. For the three locally delivered interventions, the contralateral hind limb of the same animal was assigned to a different limb-specific group, maximizing the information obtained per animal and reducing the total number of rabbits required. Specifically, seven animals contributed a left tendon to the injectable-collagen group and a right tendon to the essential oils + ASTM group; another seven contributed a left tendon to the PRP group and a right tendon to the levofloxacin-only control group. Because vitamin E + selenium is a systemic intervention, both Achilles tendons of each of the remaining seven animals were assigned to that group, yielding 14 tendons. This produced 7 tendons in each of the collagen, essential oils + ASTM, PRP and levofloxacin-only control groups, 14 in the vitamin E + selenium group, and 42 tendons in total. Assignment of treatments to limb side was fixed by protocol rather than randomized. The injected interventions (collagen, PRP) were delivered to the left limb and the comparator conditions (essential oils + ASTM, levofloxacin-only control) to the right limb in every animal. Treatment is therefore confounded with limb side in the analyzed dataset; this is acknowledged as a design limitation (Section 4).

### 2.7. Anesthesia, Analgesia and Welfare Monitoring

Tissue harvest was performed under general anesthesia induced by intramuscular ketamine hydrochloride (35 mg/kg; Ketamidor^®^ 100 mg/mL, VetViva Richter GmbH, Wels, Austria) combined with xylazine hydrochloride (5 mg/kg; Xylazin Bio 2%, Bioveta a.s., Ivanovice na Hané, Czech Republic), as described in Section 2.14. PRP blood collection and peritendinous injection were performed, like the collagen infiltration, under gentle manual restraint with local anesthesia (Section 2.8). The collagen infiltration was performed under local anesthesia with 1% lidocaine hydrochloride (Xilină 10 mg/mL; Zentiva S.A., Bucharest, Romania). Topical essential-oil application with ASTM was performed in awake animals under gentle manual restraint, as described below. Animal welfare was assessed daily by the attending veterinary staff using a structured observation record covering food and water intake, body weight, posture and gait, grooming, and behavioral signs of pain or distress (reduced activity, guarding, altered temperament); predefined thresholds for veterinary reassessment included persistent anorexia beyond 24 h, weight loss exceeding 10%, non-weight-bearing lameness, or local complications at the intervention sites. The approved protocol specifically authorized omission of post-procedural analgesia, as the interventions were limited to brief percutaneous injections; no predefined threshold was reached and no signs of pain or distress were recorded in the daily welfare assessment.

### 2.8. Platelet-Rich Plasma Preparation and Administration

PRP was prepared using a standardized single-spin protocol. Under mild restraint, 2 mL of whole blood was collected from the saphenous vein into a sterile tube containing 4% sodium citrate as anticoagulant, using a strictly aseptic technique. The sample was centrifuged at 2800 rpm for 4 min on a ROTOFIX^®^ 32 A benchtop centrifuge (Andreas Hettich GmbH & Co. KG, Tuttlingen, Germany), yielding approximately 0.4 mL of platelet-rich plasma. The entire volume was injected percutaneously under local anesthesia on the same day into the peritendinous space at the calcaneal insertion of the left Achilles tendon, with the interval between collection and injection kept under 30 min for every animal. Platelet concentration, leukocyte content and activation status of the preparation were not quantified; thus, the product could not be formally classified using the PAW system [44]. The relative centrifugal force of the 2800 rpm, four-minute spin was not recorded and the platelet yield of this protocol was not validated; so, the platelet concentration achieved relative to whole blood is unknown. This matters because the therapeutic effect of PRP in a rat Achilles tendinopathy model has been shown to be platelet-concentration-dependent [45].

### 2.9. Injectable Collagen Administration

Animals in the collagen group received a single percutaneous peritendinous infiltration of injectable porcine type I collagen (MD-Tissue^®^, Guna S.p.A., Milan, Italy; porcine-origin type I collagen; excipients: citric acid, nicotinamide, sodium chloride, water for injection). Under local anesthesia, 0.5 mL of the product was administered with a 1 mL sterile syringe at the level of the paratendon, approximately 5 mm proximal to the calcaneal insertion of the left Achilles tendon, on day 1, using a strictly aseptic technique.

### 2.10. Vitamin E and Selenium Supplementation

Animals in the vitamin E + selenium group received daily oral supplementation with Introvit-E-Selenium (Interchemie Werken De Adelaar B.V., Venray, The Netherlands), an oral solution containing 50 mg vitamin E (α-tocopheryl acetate) and 0.5 mg sodium selenite per mL. A fixed volume of 4 mL was administered orally once daily for 14 consecutive days, starting on day 1, concomitantly with levofloxacin. Relative to the mean baseline body weight of the cohort (4.11 kg), this corresponded to approximately 48.7 mg/kg/day of vitamin E and 487 µg/kg/day of sodium selenite (approximately 222 µg/kg/day of elemental selenium); selenium doses are expressed as the salt throughout unless stated otherwise. Because a fixed volume was administered rather than a body-weight-adjusted dose, actual exposure varied with animal weight (3.40–4.74 kg), from approximately 42.2 to 58.8 mg/kg/day of vitamin E and from approximately 422 to 588 µg/kg/day of sodium selenite (193–269 µg/kg/day of elemental selenium). Serum or tissue concentrations of vitamin E and selenium were not measured; therefore, no biochemical confirmation that supplementation modified antioxidant status is available. The doses were selected in accordance with previously published experimental studies of vitamin E and selenium supplementation in animal models of oxidative stress and tissue injury [28,30,31].

### 2.11. Essential Oil Formulation and Topical Application with Augmented Soft-Tissue Mobilization

The topical anti-inflammatory formulation was prepared according to a previously described essential-oil blend [46] with documented anti-inflammatory and analgesic properties, originally developed at the University of Strasbourg. The botanical identity, geographical origin, supplier, and manufacturer-reported physicochemical specifications of each constituent oil and of the vehicle are given in Table 1. The blend comprised balsam fir (*Abies balsamea*), lemon eucalyptus (*Eucalyptus citriodora*), katrafay (*Cedrelopsis grevei*), Italian immortelle (*Helichrysum italicum*), and mastic tree (*Pistacia lentiscus*). Table 1 reports the botanical identity, suppliers, and manufacturer-reported compositional specifications; the blend was compounded, per 20 mL of final preparation, from 10 µL of *Abies balsamea*, 15 µL of *Cedrelopsis grevei*, 15 µL of *Eucalyptus citriodora*, 5 µL of *Helichrysum italicum*, and 5 µL of *Pistacia lentiscus* essential oil (50 µL of essential oils in total, corresponding to a total essential-oil concentration of approximately 0.25% *v*/*v*), made up to volume with the neutral gel vehicle. Batch numbers are not reported, and no independent chromatographic analysis of the administered blend was performed; its compositional specification therefore relies on manufacturer data.

The five oils were combined with the Fitalite™ (Fagron UK Ltd., Newcastle upon Tyne, UK) gel-cream base in the quantities specified above (10 + 15 + 15 + 5 + 5 µL of essential oils per 20 mL of final preparation). The formulation was applied to the right Achilles tendon by ASTM daily for 21 consecutive days, starting on day 1. For each session, the rabbit was placed in prone position with the hind limbs elevated, awake, while a trained assistant provided gentle manual restraint. Five drops (approximately 200 µL) of the essential-oil gel were applied directly to the skin overlying the right Achilles tendon. The tendon was then massaged with steady pressure in a bidirectional, distal-to-proximal and proximal-to-distal motion along its full length for approximately 3 min per session. The applied dose was standardized as five drops (approximately 200 µL) per session, but the mobilization pressure was not standardized by force and operator reliability was not assessed; the intervention is therefore a compound one (five botanical oils, a vehicle, daily manual restraint, and daily mobilization), and no vehicle-only, oil-only, mobilization-only, or handling-matched comparator was included.

### 2.12. Levofloxacin-Only Control Group

Animals contributing tendons to the levofloxacin-only control group received only oral levofloxacin at 100 µg/kg/day for seven days, with no adjuvant therapy applied to the tendon concerned. The right Achilles tendon of each of these animals served as the untreated levofloxacin-only control; the contralateral left tendon of the same animal was assigned to the PRP group (Section 2.6). The control condition is therefore defined at the level of the tendon rather than the animal, and no sham injection or vehicle control was performed; it therefore represents untreated fluoroquinolone exposure rather than a procedure-matched comparator.

### 2.13. Ultrasonographic Follow-Up

Bilateral B-mode ultrasonographic examination of Achilles tendons was performed on day 1 (baseline, before any intervention), on day 14, and at three months (end of follow-up, immediately before euthanasia). Imaging was performed with a MyLab X5 (Esaote, Genoa, Italy) system using a linear-array transducer at 3–13 MHz, by the same experienced physician throughout, with an SL1543 linear-array probe (55 mm footprint, Esaote SpA, Genoa, Italy), in both longitudinal and transverse planes, covering the full tendon from the musculotendinous junction to the calcaneal insertion. Tendon thickness, echogenicity, fibrillar architecture, focal lesions, peritendinous fluid, and peritendon changes were recorded for every tendon using a standardized proforma; in addition, anteroposterior tendon thickness was measured in millimeters at each examination at the midportion of the tendon, recorded as the average of measurements obtained at the mid-level between the calcaneal insertion and the musculotendinous junction. No duplicate measurements were obtained, and the intra-rater test–retest reliability of the thickness measurement therefore could not be quantified; reporting the measurement level is nevertheless essential, given the marked variation of tendon thickness with distance from the calcaneal insertion (Section 3.10). Thickness was recorded for every tendon at baseline and at day 14. At the three-month examination, a thickness value was obtained in only three tendons; so, the quantitative longitudinal analysis is confined to the baseline-to-day-14 interval.

### 2.14. Euthanasia, Tissue Harvest and Macroscopic Assessment

At the end of the three-month follow-up period, the animals were deeply anesthetized with ketamine hydrochloride (Ketamidor^®^ 100 mg/mL, VetViva Richter GmbH, Wels, Austria) at 35 mg/kg and xylazine hydrochloride (Xylazin Bio 2%, Bioveta a.s., Ivanovice na Hané, Czech Republic) at 5 mg/kg, administered intramuscularly. Following confirmation of a surgical plane of anesthesia, the animals were euthanized by intravenous administration of sodium pentobarbital (Euthasol vet. 400 mg/mL, Le Vet Beheer B.V., Oudewater, The Netherlands) at 100–150 mg/kg via the marginal ear vein. The Achilles tendons were harvested by careful dissection, together with their paratendon, the calcaneal insertion, and the musculotendinous junction. After harvest, specimen thickness was measured directly with calipers at two standardized levels, 20 mm and 35 mm proximal to the calcaneal insertion, and ultrasonography was repeated post mortem on the harvested specimen at the 20 mm level. In the four groups other than vitamin E + selenium, the plantaris tendon was retained en bloc and is therefore included in these ex vivo measurements.

Macroscopic appearance was assessed and recorded using a standardized proforma immediately before harvest, with peritendinous adhesions and hyperaemia as the findings of interest. Every tendon was examined; absence of an entry denotes no abnormality detected rather than missing data.

Throughout the study, no clinically significant changes in body weight, food intake, or general behavior were observed, and no overt systemic or adverse behavioral effects attributable to any intervention were recorded. Local tissue findings at dissection (peritendinous adhesions and hyperaemia; Section 3.7) are reported separately and are not subsumed under this statement, as some may represent intervention-associated local reactions.

### 2.15. Histological Processing

**Figure 2:**
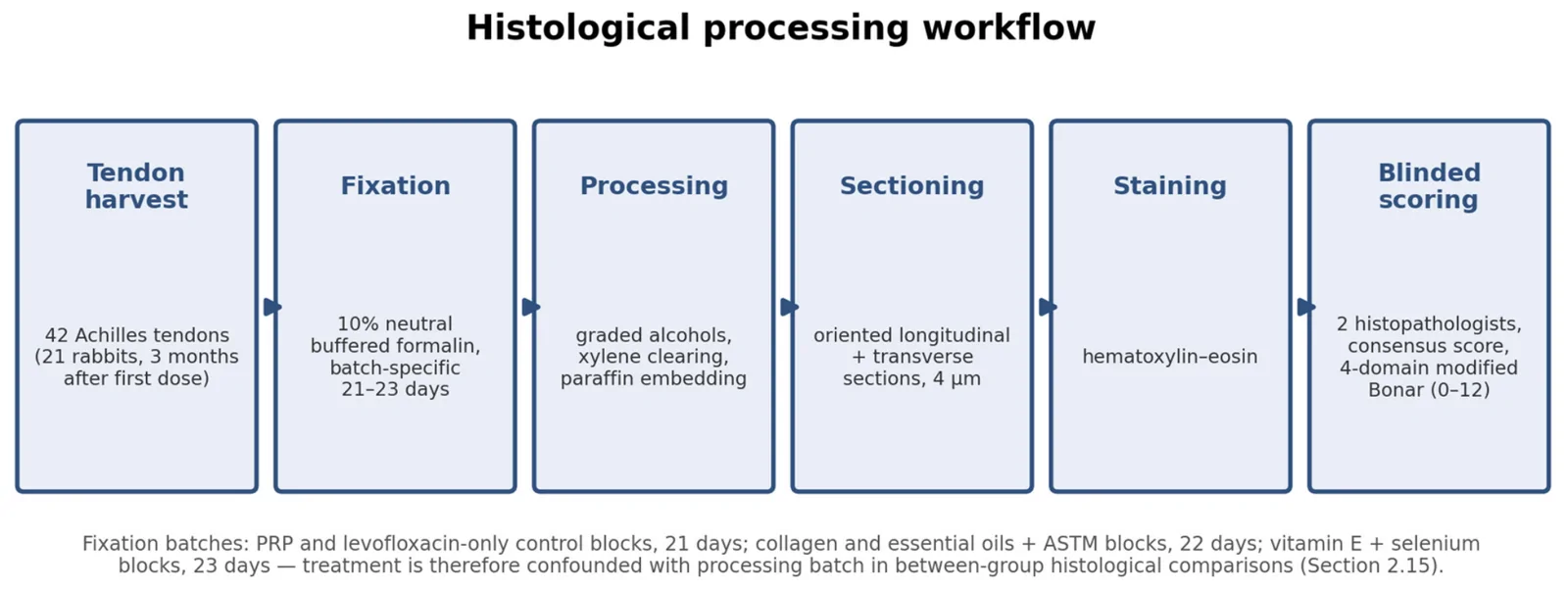
Overview of the histological processing workflow for the 42 Achilles tendon specimens, from harvest to blinded consensus scoring.

Specimens were fixed in 10% neutral buffered formalin for a group-specific duration determined by processing batch: 21 days for the PRP and levofloxacin-only control blocks, 22 days for the collagen and essential oils + ASTM blocks, and 23 days for the vitamin E + selenium blocks. Then, the specimens were dehydrated through graded alcohols, cleared in xylene, and embedded in paraffin. Because fixation duration and processing batch were group-specific, treatment is confounded with processing batch in all between-group histological comparisons; the two within-animal paired comparisons were processed within a single batch and fixation interval. Future studies should assign specimens from all groups randomly across processing batches and standardize the fixation interval. Each block contained an oriented longitudinal section running from the calcaneal insertion to the musculotendinous junction, followed by a transverse section through the same specimen. Sections of 4 µm were cut from each block, mounted on standard glass slides, and stained with hematoxylin–eosin. The histological processing workflow is summarized in Figure 2.

### 2.16. Histopathological Evaluation and Modified Bonar Scoring

Histopathological assessment was performed on one central longitudinal section per tendon, examined in its entirety; grading of each domain reflected the most prominent changes observed within that predefined central section.

Tendons were graded using a modified Bonar system adapted from the original semi-quantitative Bonar score [47] and its subsequent revision, which introduced a separate cellularity domain [48]. The scoring form provided for six domains: tenocyte morphology, cellularity, vascularity, paratendinous fibrosis, ground substance content, and collagen fiber organization. Scoring of the ground-substance and collagen-organization domains was not carried out for the series, and these two domains were therefore excluded from analysis. The analyzed composite comprised the four completed domains: tenocyte morphology, cellularity (tenocyte number), vascularity, and paratendinous fibrosis, the latter reflecting the peritendinous reaction relevant to this model. Each domain was scored from 0 (normal) to 3 (markedly abnormal), and the composite score (range 0–12) was calculated as the sum of the four domain scores. Because this composite departs from the classic Bonar score, absolute values are not directly comparable with published Bonar data, and the omission of the two matrix domains may under-capture the extracellular-matrix changes characteristic of fluoroquinolone exposure.

All slides were evaluated independently by two histopathologists who were blinded to group allocation. Where the two observers disagreed, the slide was assessed independently by a third histopathologist, followed by committee discussion until consensus was reached; the reconciled consensus score was used for analysis. Only the reconciled consensus score was retained for each tendon; so, inter-observer agreement cannot be quantified. This prevents estimation of weighted kappa or intraclass correlation coefficients for the primary outcome and is acknowledged as a limitation; future studies should retain the initial independent scores, as in animal histopathological scoring protocols that incorporate inter-rater agreement from the outset [49].

### 2.17. Data Handling

Histopathological grades, macroscopic findings, ultrasonographic descriptors, and thickness measurements were recorded for every tendon and linked by the animal identifier and limb side. Body weight was recorded at baseline, and food intake and general behavior were monitored daily as tolerability measures. No data were imputed and no tendon was excluded from analysis; the only missing quantitative data were the three-month in vivo thickness measurements, obtained in 3 of 42 tendons only (Section 2.13). The complete dataset for all 42 tendons is provided as Supplementary Table S1.

### 2.18. Statistical Analysis

Continuous variables are summarized as mean ± standard deviation where approximately symmetrical and as median with interquartile range otherwise; ordinal variables, namely the individual modified Bonar domain grades and the composite score, are summarized as median with interquartile range. Categorical findings are reported as counts and percentages of tendons.

Because the composite score is an ordinal variable with a restricted range and a pronounced floor effect, non-parametric methods were used for the primary analysis. Differences between the five groups in the composite score and in each individual domain were tested with the Kruskal–Wallis test, followed by Dunn’s post hoc test with Bonferroni correction for multiple comparisons. The magnitude of between-group differences was expressed as epsilon-squared. The experimental unit differed between interventions; in the two locally delivered paired comparisons, the tendon was the treated unit within an animal block, whereas for the systemic vitamin E + selenium intervention, the animal was the experimental unit and its two tendons were duplicate observations of the same exposure. Analyses treating all 42 tendons as independent therefore overstate the effective sample size, particularly for the systemic group, and all five-group comparisons are reported as exploratory and descriptive only. As sensitivity analyses, the principal analyses were repeated at the animal level by averaging the two tendons of each animal (body weight–composite score correlation, n = 21; within-group thickness change for vitamin E + selenium, n = 7). Exact signed-rank confidence intervals were calculated for the within-animal median differences, and a Benjamini–Hochberg false-discovery-rate correction was applied across the fifteen tests bearing directly on treatment-group comparisons.

The contralateral-limb design permitted two comparisons internal to each animal and therefore free of between-animal variation: injectable collagen versus essential oils + ASTM, and PRP versus the levofloxacin-only control. These were analyzed with the Wilcoxon signed-rank test, and *p*-values are reported both unadjusted and Bonferroni-adjusted for the two paired comparisons performed. Left–right symmetry within the vitamin E + selenium group, in which both limbs received the same systemic intervention, was assessed with the same test together with Spearman correlation between paired limbs.

To quantify clustering of tendons within animals, a linear mixed-effects model of the composite score was fitted with treatment group as a fixed effect and animal as a random intercept; the intraclass correlation coefficient was derived from the resulting variance components.

Ultrasonographic thickness was compared between groups with the Kruskal–Wallis test and within tendons over time with the Wilcoxon signed-rank test. Because baseline thickness was not balanced across groups, day-14 thickness was additionally analyzed by analysis of covariance with baseline thickness as covariate and treatment group as factor, the group effect being tested by partial F-test against the baseline-only model. Regression to the mean was assessed by correlating baseline thickness with subsequent change. Agreement between post-mortem ultrasonography and direct caliper measurement of the same specimen was assessed by the Bland–Altman method, reporting mean bias and 95% limits of agreement, with the systematic component tested by Wilcoxon signed-rank test.

Associations between continuous or ordinal variables were quantified by Spearman rank correlation, and comparisons between two independent groups by the Mann–Whitney U test. The distribution of categorical findings across the five groups was assessed by a permutation test based on the chi-squared statistic, chosen because expected cell counts were small, and pairwise categorical comparisons used Fisher’s exact test with odds ratios.

All tests were two-sided and a *p*-value below 0.05 was regarded as nominally significant. Because this was an exploratory pilot study, no comparison was designated as confirmatory and, except where explicitly stated, *p*-values are not adjusted for the total number of analyses performed. All *p*-values are therefore interpreted descriptively, as estimates of the direction and consistency of an effect rather than as tests of a prespecified hypothesis. No interim analysis was performed.

All statistical analyses were performed using Microsoft Excel (version 16.83, Microsoft 365 for macOS), GraphPad Prism (version 10.1.2 for macOS), and R Studio (version 2025.05.0+496). The significance threshold was set at *p* < 0.05 throughout, unless otherwise specified.

## 3. Results

### 3.1. Animals, Tolerability and Specimen Yield

All 21 rabbits completed the three-month protocol. There were no deaths, no premature terminations, and no exclusions; thus, all 42 Achilles tendons entered the analysis (7 tendons each in the essential oils + ASTM, injectable collagen, PRP and levofloxacin-only control groups, and 14 in the vitamin E + selenium group). Throughout the study, no clinically significant changes in body weight, food intake, or general behavior were observed, and no adverse effect attributable to levofloxacin or to any adjuvant intervention was recorded. Local macroscopic findings at dissection, some of which may represent intervention-associated tissue reactions, are reported in Section 3.7. Baseline body weight ranged from 3.40 to 4.74 kg (mean 4.11 kg). Because the two tendons of an animal share its body weight, the association with the composite histopathological score was assessed at the animal level (n = 21, averaging the two tendon scores of each animal). Body weight was not significantly associated with the composite score (Spearman *ρ* = 0.350, *p* = 0.12), although a sample of 21 animals cannot firmly exclude a weight effect. Every tendon underwent macroscopic assessment at dissection and bilateral ultrasonographic examination at all three timepoints, with quantitative thickness recorded at baseline and day 14, and a complete four-domain histopathological score was obtained for every specimen; no data were missing for these prespecified assessments and none were imputed, although a reliable quantitative thickness value at the three-month examination was obtained in only 3 of 42 tendons (Section 3.9). The complete per-tendon dataset is provided as Supplementary Table S1.

### 3.2. Composite Modified Bonar Score

Composite modified Bonar scores were low across the whole series, indicating only mild histopathological change three months after fluoroquinolone exposure. Across all 42 tendons, the median composite score was 4.0 (IQR 2.0–5.8) out of a maximum possible 12; 20 of 42 tendons (48%) scored 3 or less, 5 tendons (12%) scored 0, and no tendon exceeded 8 of 12.

**Figure 3:**
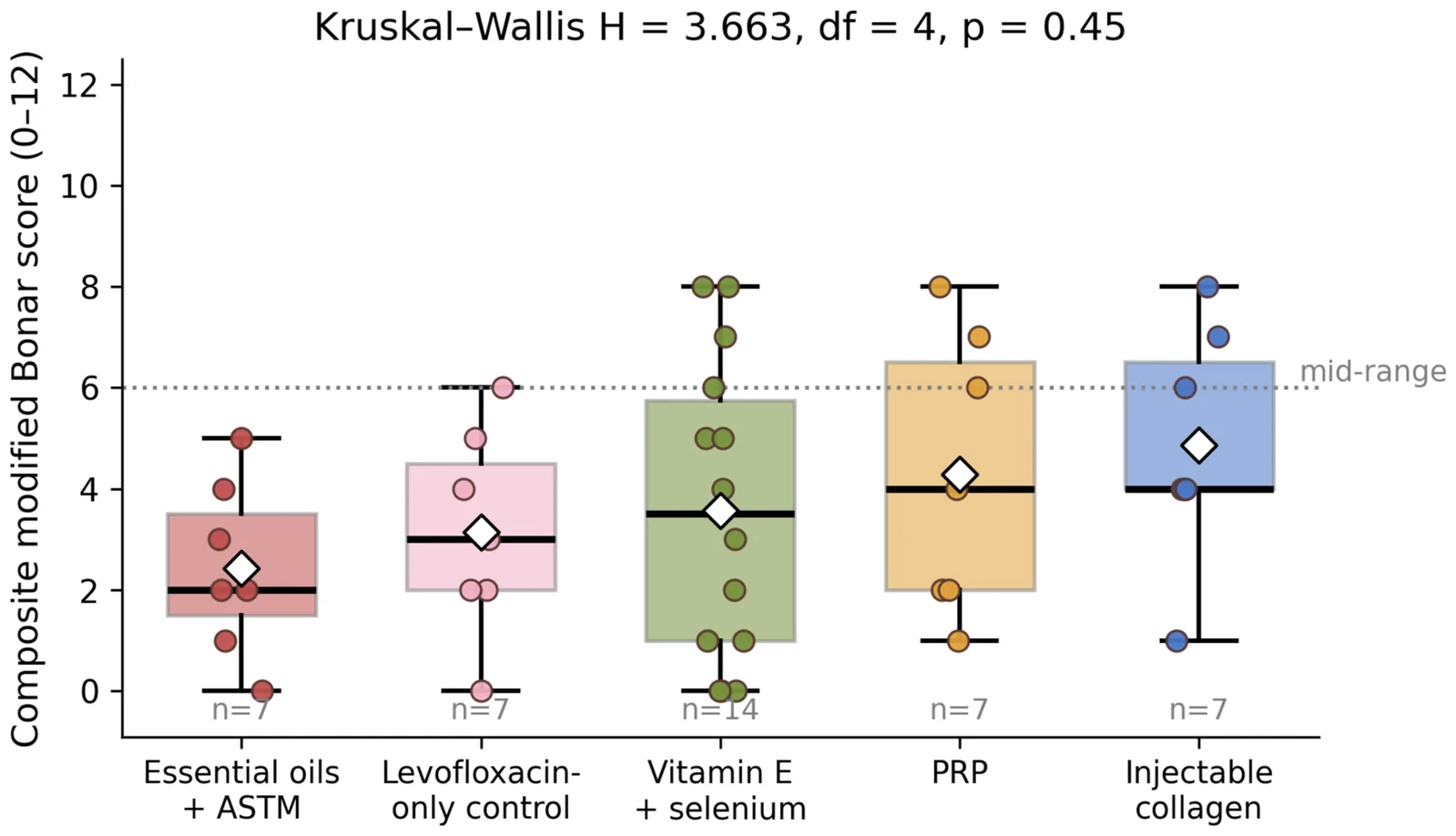
Composite modified Bonar score by experimental group. Boxes show the median and interquartile range, whiskers the range, diamonds the group mean, and individual points every tendon analyzed. The dotted line marks the mid-point of the 0–12 scale. All groups lie in the lower half of the scale, illustrating the floor effect that constrains this comparison.

Group medians are given in Table 2 and the distribution is shown in Figure 3. Median [IQR] composite scores were 2.00 [1.50–3.50] for essential oils + ASTM, 3.00 [2.00–4.50] for the levofloxacin-only control, 3.50 [1.00–5.75] for vitamin E + selenium, 4.00 [2.00–6.50] for PRP, and 4.00 [4.00–6.50] for injectable collagen. The Kruskal–Wallis test showed no significant difference between the five groups (H = 3.663, df = 4, *p* = 0.45), and the corresponding effect size was negligible (*ε*^2^ ≈ 0). All Dunn post hoc comparisons were non-significant after Bonferroni correction, the smallest adjusted *p*-value being 0.81 for essential oils + ASTM versus injectable collagen. Given the allocation structure described in Section 2.6, this five-group comparison is descriptive and exploratory rather than confirmatory (Section 2.18).

Contrary to the study hypothesis, the levofloxacin-only control group did not show the greatest histopathological change. Both injectable arms scored numerically higher than the control, and only the essential oils + ASTM group scored numerically lower. Considered as the proportion of tendons showing moderate or marked change in at least one domain (grade ≥ 2), the same ordering was seen: 3 of 7 (43%) for essential oils + ASTM, 5 of 7 (71%) for control, 7 of 14 (50%) for vitamin E + selenium, and 6 of 7 (86%) for both PRP and injectable collagen, although this distribution did not reach significance (*p* = 0.23).

### 3.3. Individual Histopathological Domains

**Figure 4:**
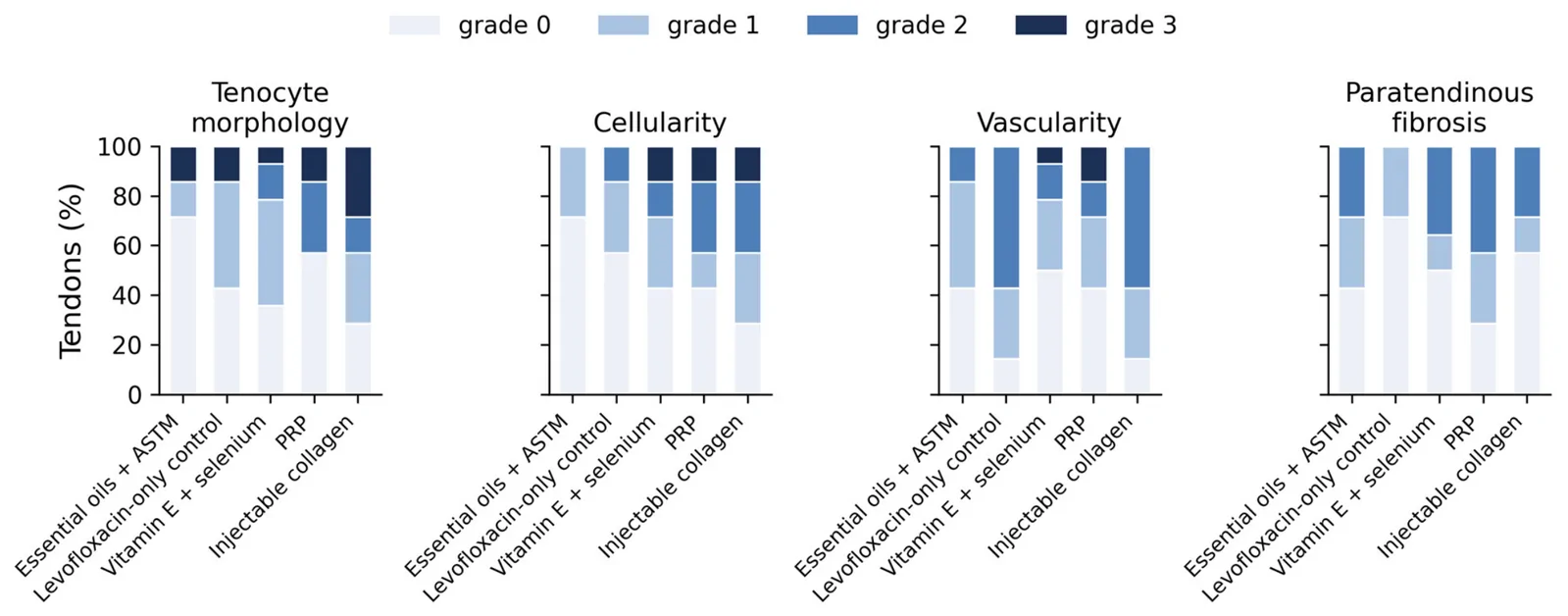
Distribution of domain grades by group. Each bar shows the percentage of tendons in that group assigned grade 0, 1, 2, or 3 for the domain indicated. The predominance of grades 0 and 1 in every group and every domain illustrates the limited severity achieved by the model.

No individual domain differed significantly between the five groups (Table 3, Figure 4). Vascularity was the most consistently elevated domain across the series and was highest in the levofloxacin-only control and injectable collagen groups (median 2.0 in both), whereas the vitamin E + selenium group had the lowest vascularity scores (median 0.5). The two cellular domains, tenocyte morphology and cellularity, were highest in the injectable arms and lowest in the essential oils + ASTM group, in which the median for both was 0. Paratendinous fibrosis showed the opposite pattern, being highest in the PRP and essential oils + ASTM groups (median 1.0 in both) and lowest in the untreated levofloxacin-only control and injectable collagen groups (median 0).

Tenocyte morphology and cellularity were strongly correlated across the series (Spearman *ρ* = 0.750, *p* < 0.001), and cellularity correlated weakly with vascularity (*ρ* = 0.308, *p* = 0.047). Paratendinous fibrosis was independent of all three intratendinous domains (all *p* > 0.5), consistent with it capturing a peritendinous rather than an intratendinous process.

### 3.4. Within-Animal (Contralateral Limb) Comparisons

Because three of the interventions were delivered locally to one hind limb, with the contralateral limb of the same animal assigned to a different group, two comparisons could be made within animals and are therefore free of between-animal variation. These are the only analyses in this study that fully reflect the experimental design, and they are reported alongside the between-group analysis.

**Figure 5:**
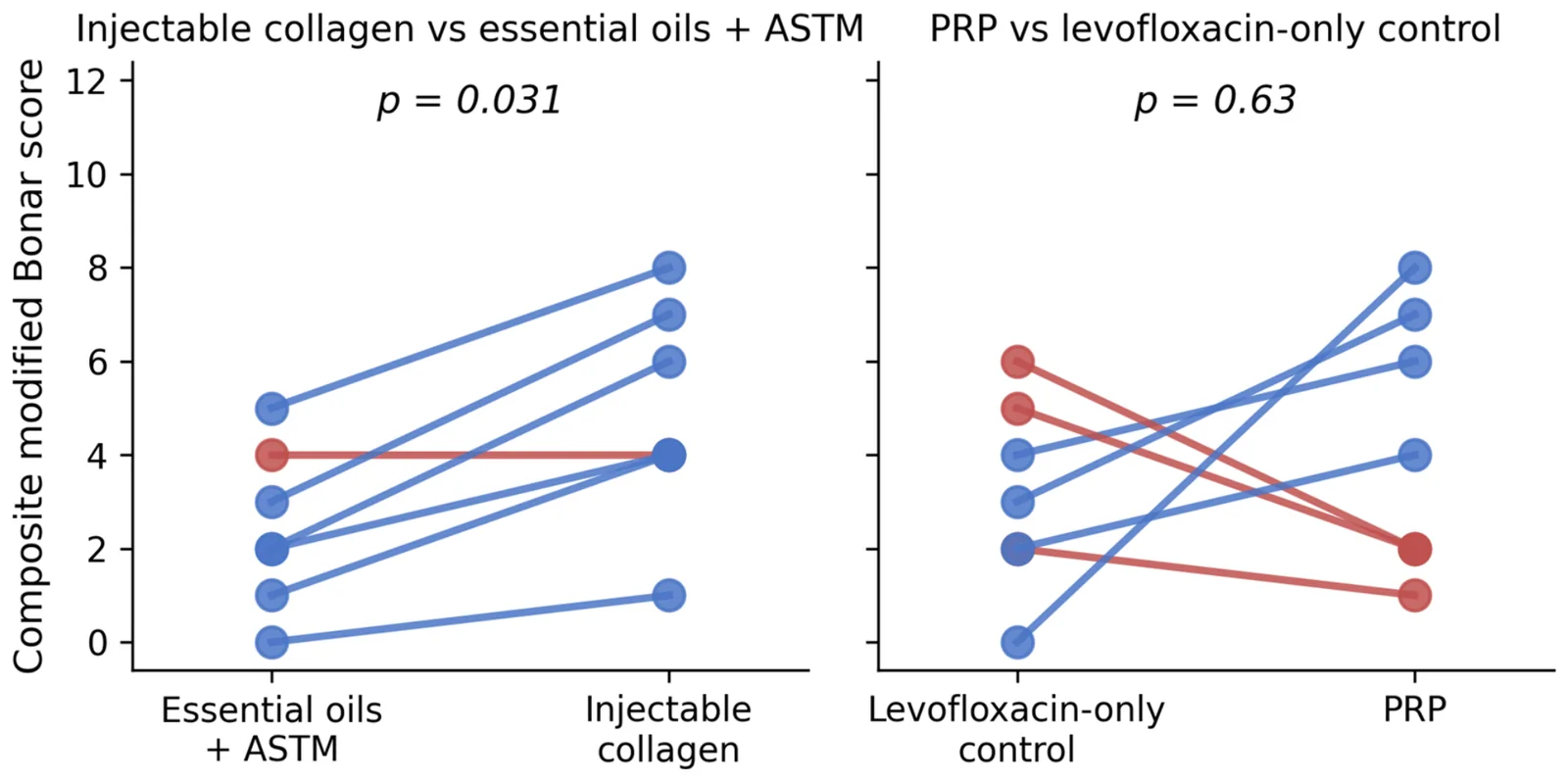
Within-animal comparisons of the composite modified Bonar score. Each line connects results for the two tendons of a single animal. Blue lines indicate a higher score in the group on the right, red lines a higher score on the left. (**Left panel**): injectable collagen scored higher than essential oils + ASTM in six of seven animals and equal in the seventh. (**Right panel**): PRP versus levofloxacin-only control showed no consistent direction.

In the seven animals that contributed one tendon to the injectable collagen group and one to the essential oils + ASTM group, the collagen-treated tendon scored higher in six of seven pairs and equal in the seventh, with a median within-animal difference of +3.0 points (Wilcoxon signed-rank test, *p* = 0.031; exact 95.3% signed-rank confidence interval for the difference, +1.0 to +4.0 points). In the seven animals that contributed one tendon to the PRP group and one to the levofloxacin-only control, the PRP-treated tendon scored higher in four of seven pairs, with a median difference of +2.0 points, but the comparison was not significant (*p* = 0.63; exact 95.3% confidence interval, −3.0 to +5.0 points). The individual pairs are plotted in Figure 5 and summarized in Table 4.

The collagen versus essential-oil difference is the only comparison in this pilot to reach nominal significance. In an exploratory pilot of this size it should be interpreted with particular caution, for three reasons. It is a comparison between two active treatments rather than against the untreated control; it rests on seven pairs; and it is one of two paired tests performed, so it does not survive Bonferroni correction for those two comparisons (adjusted *p* = 0.062). Under the Benjamini–Hochberg false-discovery-rate correction across the fifteen treatment-related tests performed in this study (Section 2.18), this comparison likewise did not remain below the 0.05 threshold (adjusted *p* ≈ 0.06). At the level of individual domains, the same direction was seen for the three intratendinous domains without reaching significance (tenocyte morphology *p* = 0.38, cellularity *p* = 0.13, vascularity *p* = 0.25), while paratendinous fibrosis showed no difference (*p* = 0.91), indicating that the composite difference was driven by diffuse intratendinous change rather than by any single domain.

In the vitamin E + selenium group, in which both limbs of each animal received the same systemic intervention, the left and right tendons of the same animal did not differ systematically (median 3.0 versus 5.0, *p* = 0.53) and were not correlated with one another (*ρ* = −0.431, *p* = 0.33), indicating substantial within-animal variability in the histopathological response.

### 3.5. Between-Animal Variance

A linear mixed-effects model of the composite score with treatment group as a fixed effect and animal as a random effect was fitted to quantify clustering within animals. The estimated between-animal variance component was effectively zero (intraclass correlation ≈ 0), and the model was singular at that boundary, indicating that tendons from the same animal were no more alike than tendons from different animals. Fixed-effect estimates relative to the levofloxacin-only control were −0.71 points for essential oils + ASTM (*p* = 0.60), +0.43 for vitamin E + selenium (*p* = 0.71), +1.14 for PRP (*p* = 0.40) and +1.71 for injectable collagen (*p* = 0.20), reproducing the ordering seen in the unadjusted analysis. An intraclass correlation estimated at the zero boundary from a singular model fitted to 21 animals should not, however, be interpreted as evidence that tendons from the same animal are biologically independent; with a bounded ordinal outcome, a pronounced floor effect and a dataset of this size, a boundary estimate may reflect limited information or model misspecification rather than a true absence of clustering. The tendon-level five-group analyses are therefore regarded as exploratory, and the within-animal paired comparisons remain the analyses most consistent with the design.

### 3.6. Distribution of Scores Across the Series

**Figure 6:**
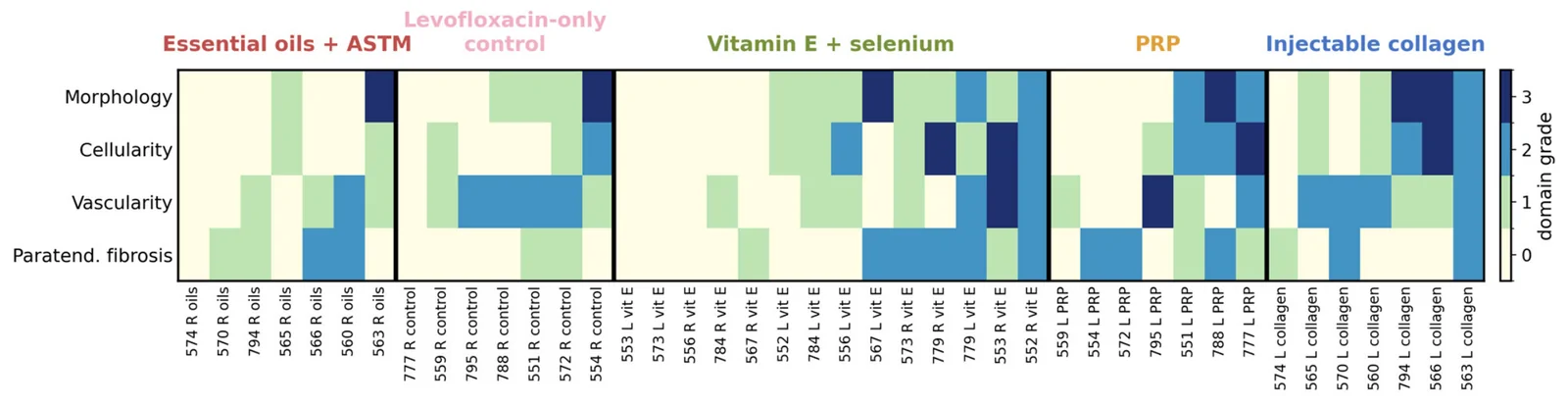
Per-tendon heat map of the four scored modified Bonar domains for all 42 specimens, grouped by treatment and ordered within group by ascending composite score. Darker cells indicate higher grades. Vertical black lines separate the experimental groups; tendon identifiers combine the animal microchip number, limb side, and group.

Figure 6 displays every tendon and every domain in the series, ordered by group and within group by composite score. Three features are apparent. First, high scores are scattered rather than clustered; each of the five groups contains at least one tendon scoring 4 or less, and four of the five contain a tendon scoring 6 or more. Second, the vitamin E + selenium group shows the widest dispersion, spanning the full observed range from 0 to 8, which reflects both its larger size and the within-animal variability noted above. Third, no group shows a coherent pattern of change across all four domains, and no tendon reached grade 3 in more than two domains simultaneously.

### 3.7. Macroscopic Findings at Dissection

**Figure 7:**
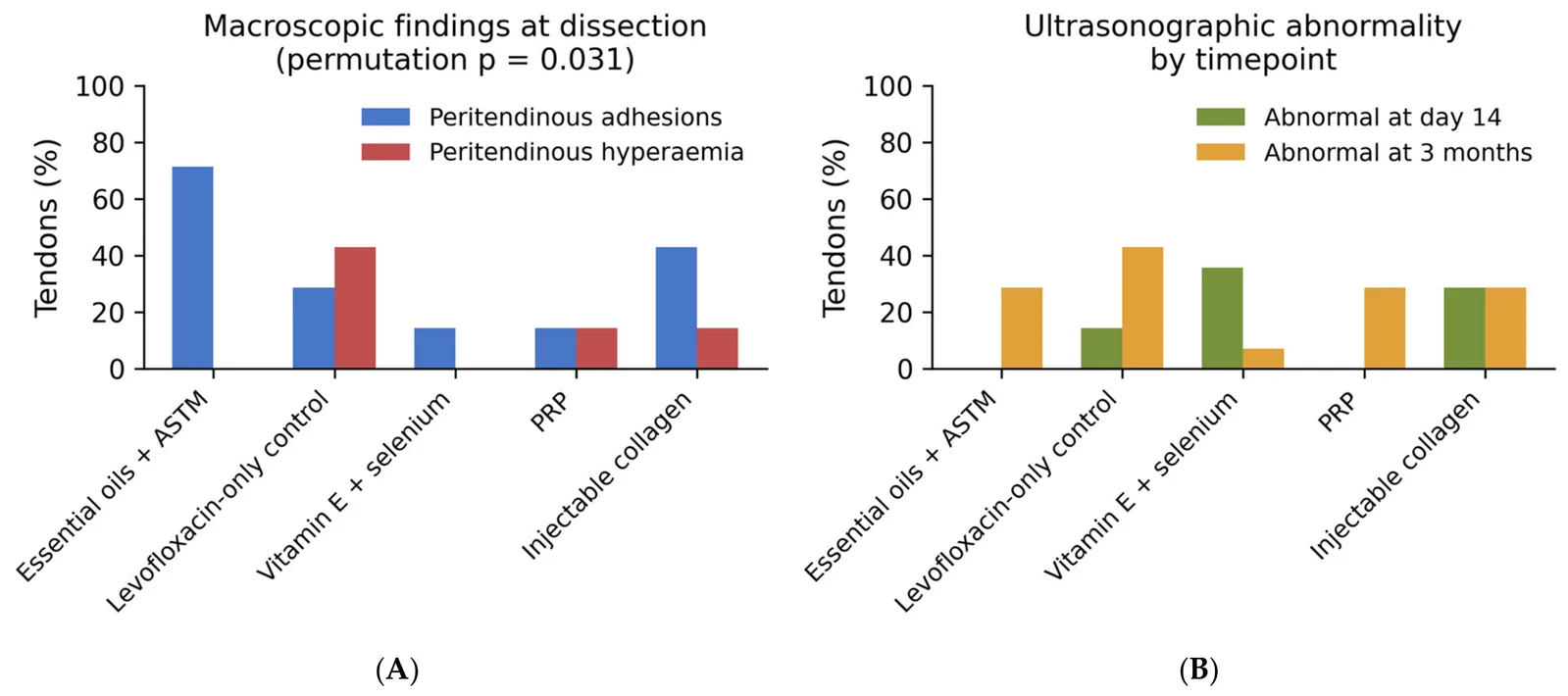
Macroscopic and ultrasonographic findings by group. (**A**) Percentage of tendons showing peritendinous adhesions or hyperaemia at dissection; the overall distribution differed between groups (permutation *p* = 0.031). (**B**) Percentage of tendons with an abnormal ultrasonographic appearance at day 14 and at three months. All tendons were normal at baseline.

Macroscopic assessment was completed for all 42 tendons. An abnormality was recorded in 18 of 42 (43%): peritendinous adhesions in 13 tendons (31%) and peritendinous hyperaemia in 5 (12%), with no tendon showing both. The distribution differed between groups (permutation test on the 5 × 2 table, *p* = 0.031; Table 5, Figure 7A). Adhesions were most frequent in the essential oils + ASTM group (5 of 7, 71%) and least frequent in the vitamin E + selenium group (2 of 14, 14%); the direct comparison between these two groups was significant (Fisher exact *p* = 0.017, odds ratio 15.0). Hyperaemia occurred only in the levofloxacin-only control (3 of 7), PRP, and injectable collagen groups (1 of 7 each), and in two of these five tendons, it was localized specifically to the calcaneal insertion, which is the site at which the injections were delivered.

Pooling the three locally treated groups against the systemically treated and untreated groups did not itself reach significance (11 of 21 versus 7 of 21, Fisher exact *p* = 0.35), indicating that the group differences in macroscopic appearance were driven more by the repeated mechanical stimulus of the essential oils + ASTM protocol and by the injections at the calcaneal insertion than by local treatment as a category. These categorical analyses also treat tendons from the same animal as independent observations. Like the paired collagen comparison, the overall macroscopic-finding distribution (nominal *p* = 0.031) does not remain below the conventional threshold once the multiplicity of treatment-group comparisons performed in this study is taken into account, and it should be interpreted with the same caution.

### 3.8. Ultrasonographic Findings

Bilateral ultrasonography was performed on day 1, day 14, and at three months. All tendons were normal at baseline. An abnormality was documented at one or both follow-up timepoints in 21 of 42 tendons (50%): in 8 tendons at day 14 and in 10 at three months, with 3 tendons abnormal at both. Group frequencies were 3 of 7 for essential oils + ASTM, 4 of 7 for the levofloxacin-only control, 6 of 14 for vitamin E + selenium, 4 of 7 for PRP, and 4 of 7 for injectable collagen (Figure 7B). Notably, five of the eight early (day 14) abnormalities occurred in the vitamin E + selenium group, whereas abnormalities in the other groups were predominantly detected at three months.

The most frequent descriptor was fibrillar disorganization or heterogeneous fiber architecture (8 tendons), followed by peritendon or paratendon change (6), hyperechogenicity (5), fusiform thickening (5), non-uniform hypoechoic zones (4), focal fiber discontinuity (3), and peritendinous fluid (1). Because only structured qualitative descriptors were recorded and the descriptors themselves were recorded qualitatively, they are reported descriptively here; the accompanying quantitative thickness measurements are analyzed in Section 3.9.

### 3.9. Quantitative Ultrasonographic Measurements

Achilles tendon thickness was measured in vivo in every tendon at baseline and at day 14; measurements at three months were obtained in only three tendons and are not analyzed. Baseline thickness was not uniform across groups (Kruskal–Wallis *p* = 0.001): tendons in the vitamin E + selenium group were thinner at baseline (median 5.20 mm, IQR 4.70–5.62) than those in the other four groups (medians 6.00–6.20 mm). Because the vitamin E + selenium group comprised a separate set of seven animals, this represents an animal-level difference present before any intervention; it is addressed, but cannot be fully corrected, in the analyses below.

**Figure 8:**
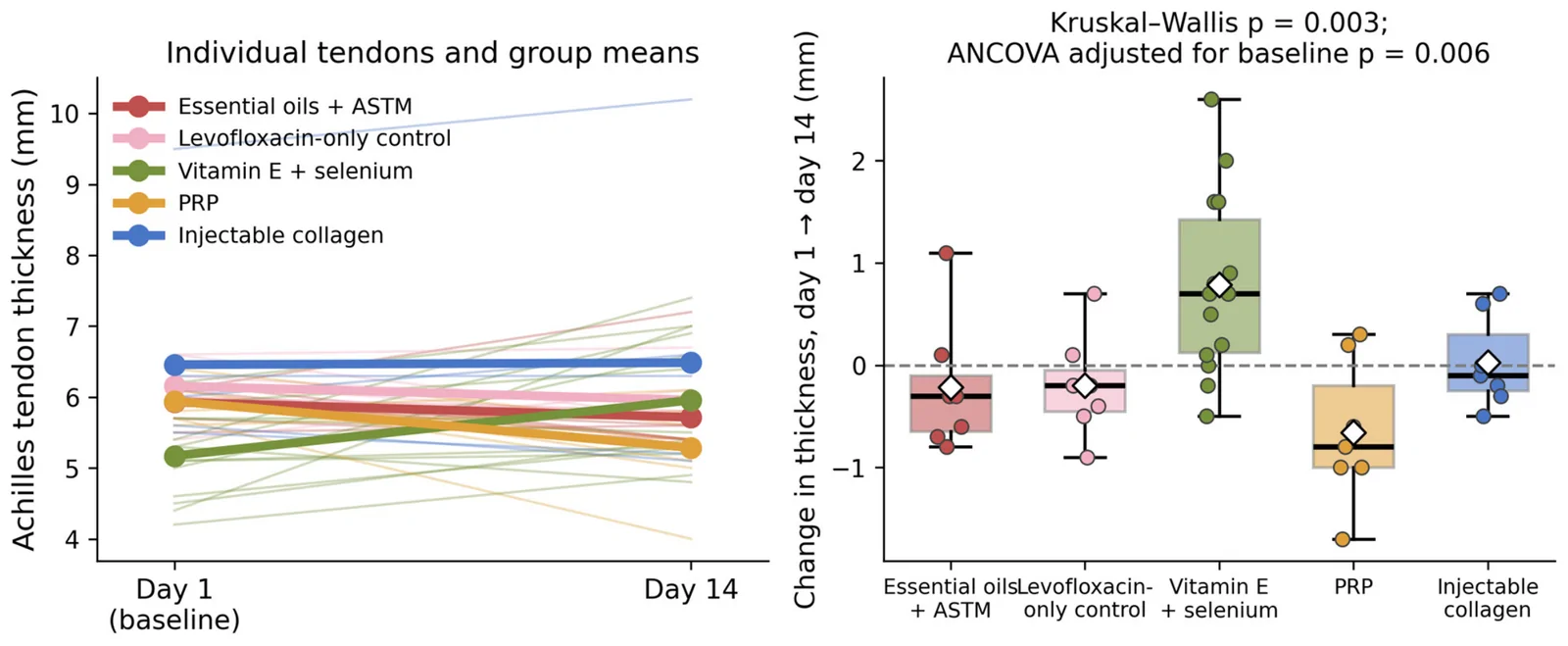
In vivo tendon thickness. (**Left**): individual tendons (faint lines) and group means (bold) between baseline and day 14. (**Right**): distribution of the change in thickness by group, with the dashed line at zero. Only the vitamin E + selenium group increased in thickness.

Between baseline and day 14 the groups diverged (Table 6, Figure 8). Thickness increased in the vitamin E + selenium group (median +0.70 mm, +14.1%; within-group Wilcoxon *p* = 0.007) and decreased or was unchanged in the other four groups: −0.80 mm (−12.5%) for PRP, −0.30 mm (−5.3%) for essential oils + ASTM, −0.20 mm (−3.3%) for the levofloxacin-only control, and −0.10 mm (−1.6%) for injectable collagen. The between-group difference was significant for both absolute and relative change (Kruskal–Wallis *p* = 0.003 for both). In the animal-level sensitivity analysis of the vitamin E + selenium group (n = 7 animals, averaging the two tendons of each animal), the within-group increase persisted (median change +0.50 mm, exact Wilcoxon *p* = 0.031, exact 95.3% confidence interval +0.10 to +1.72 mm).

**Figure 9:**
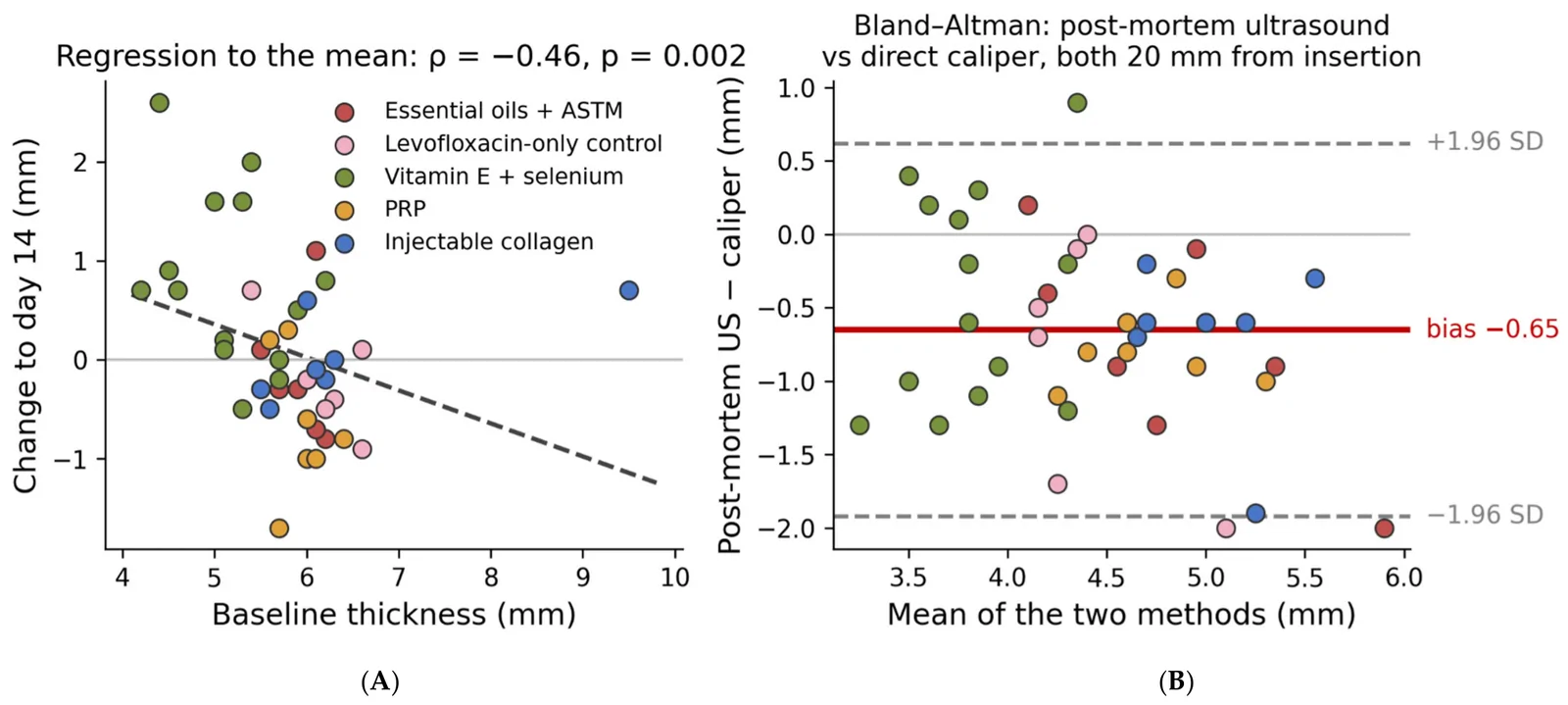
(**A**) Baseline thickness against subsequent change, showing regression to the mean across the series. (**B**) Bland–Altman comparison of post-mortem ultrasonographic measurement against direct caliper measurement of the same specimen, both taken 20 mm from the calcaneal insertion. The solid line is the mean bias and the dashed lines are the 95% limits of agreement.

Two competing explanations were examined. First, regarding regression to the mean, baseline thickness was inversely correlated with subsequent change across the series (Spearman ρ = −0.46, *p* = 0.002; Figure 9A); so, the thinner vitamin E tendons would be expected to show the largest apparent increase. Second, a genuine group effect was considered. To separate these possibilities, an analysis of covariance of day-14 thickness with baseline thickness as covariate was performed. Baseline thickness was a strong predictor (β = 0.94, *p* = 0.001), but the group effect persisted after adjustment (F(4,36) = 4.35, *p* = 0.006), with the vitamin E + selenium group +0.93 mm relative to the levofloxacin-only control (*p* = 0.017) and no other group differing significantly. Regression to the mean therefore does not fully account for the finding, although the baseline imbalance means it cannot confidently be attributed to the intervention. Two further caveats apply. First, the analysis of covariance treats all 42 tendons as independent and allocates fourteen observations to the seven systemically treated animals; therefore, its *p*-values are anticonservative with respect to the true number of experimental units. Second, because the vitamin E + selenium group coincided exactly with a separate animal cohort, statistical adjustment cannot distinguish a treatment effect from a cohort effect. Under the Benjamini–Hochberg correction described in Section 2.18, the thickness-related findings (the day-14 between-group differences, the baseline-adjusted group effect and the within-group vitamin E + selenium change) remained below an adjusted *p* of 0.05.

The two within-animal comparisons showed no significant difference in thickness change: injectable collagen versus essential oils + ASTM, median difference +0.30 mm (*p* = 0.63); PRP versus levofloxacin-only control, −0.40 mm (*p* = 0.078).

Critically, the change in tendon thickness bore no relationship to the histopathological result for the same tendon (ρ = −0.02, *p* = 0.91 against the composite score; ρ = −0.152, *p* = 0.34 against vascularity; ρ = 0.030, *p* = 0.85 against paratendinous fibrosis), nor did baseline thickness (ρ = 0.022, *p* = 0.89).

### 3.10. Ex Vivo Specimen Measurements and Method Agreement

After harvest, thickness was measured directly on the specimen 20 mm and 35 mm from the calcaneal insertion, and by ultrasonography post mortem at the 20 mm level. Tendons were substantially thicker at the proximal level: median 8.35 mm at 35 mm versus 4.80 mm at 20 mm (Wilcoxon *p* < 0.001), confirming the expected proximal taper and indicating that the level of measurement must be standardized in any future protocol.

**Figure 10:**
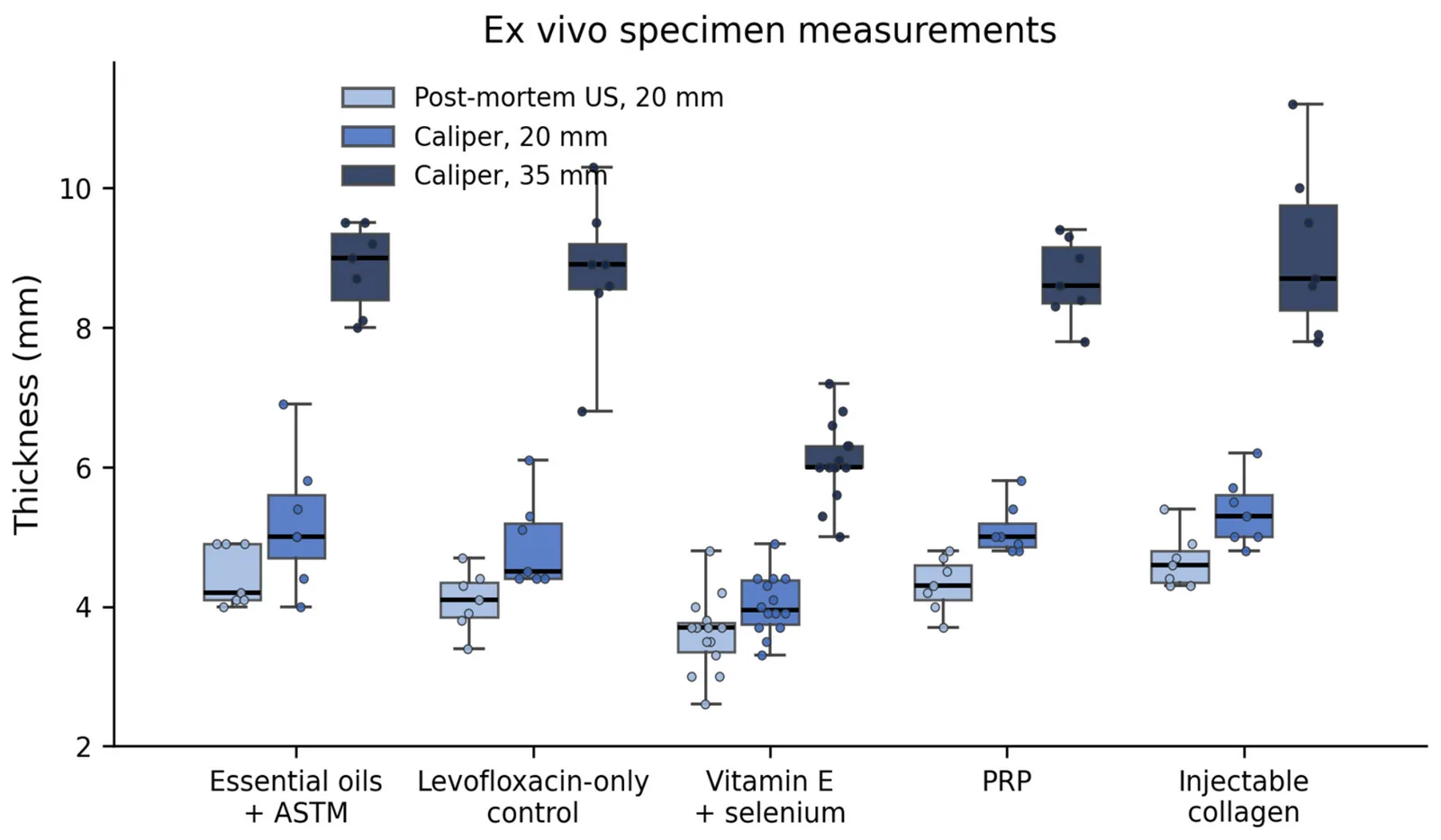
Ex vivo specimen measurements by group: post-mortem ultrasonography at 20 mm from the calcaneal insertion, and direct caliper measurement at 20 mm and 35 mm. Boxes show the median and interquartile range, whiskers show the range, and individual points indicate every specimen. The uniformly lower values in the vitamin E + selenium group reflect the exclusion of the plantaris tendon from those specimens.

All three ex vivo measures were lower in the vitamin E + selenium group than in the other four groups: 3.95 mm versus 4.50–5.30 mm at the 20 mm caliper level, and 6.00 mm versus 8.60–9.00 mm at 35 mm. The proximal-to-distal difference was likewise smaller in that group (2.00 mm versus 3.50–4.10 mm). This pattern is almost certainly a specimen-composition artefact rather than a treatment effect; in the other four groups, the plantaris tendon was harvested en bloc with the Achilles tendon and was therefore included in the caliper measurement, whereas in the vitamin E + selenium group, the Achilles tendon was measured alone. Ex vivo measurements are consequently not comparable between the vitamin E + selenium group and the remaining groups and should not be interpreted as evidence of a treatment effect. For this reason, and because the group differences are determined by specimen composition rather than by treatment, no between-group inferential statistics are presented for the ex vivo measurements (Table 7, Figure 10); they are retained for methodological completeness only, to document the measurement properties of the two techniques.

Comparison of the two measurement methods applied to the same specimens at the same level showed a systematic difference. Post-mortem ultrasonography underestimated the direct caliper measurement by a mean of 0.65 mm (Wilcoxon *p* < 0.001), with 95% limits of agreement of −1.92 to +0.62 mm and a correlation of ρ = 0.62 (*p* < 0.001; Figure 9B). Agreement between the in vivo day-14 measurement and the post-mortem measurement of the same tendon was poor (ρ = 0.21, *p* = 0.18), as expected given the three-month interval and the loss of physiological loading and perfusion after death. None of the three ex vivo measures correlated with the composite histopathological score (all *p* ≥ 0.13).

### 3.11. Agreement Between Assessment Modalities

The three assessment modalities did not converge. Composite histopathological scores were no higher in tendons with an abnormal ultrasound than in those with a normal ultrasound (median 4.0 versus 3.0, Mann–Whitney *p* = 1.00), nor in tendons with a macroscopic abnormality compared with those without (*p* = 0.89). Because abnormalities recorded at day 14 were not temporally matched to histology obtained at three months, the comparison was repeated using only the ten tendons whose abnormality was documented at the three-month examination; composite scores again did not differ (median 4.0 versus 3.5, Mann–Whitney *p* = 0.69). Even the most closely matched pairing—macroscopically visible peritendinous adhesions and the histopathological paratendinous fibrosis grade—showed no relationship (median grade 1.0 versus 0.0, *p* = 0.77). In this dataset, therefore, no correspondence between the recorded imaging or gross findings and the histopathological grade of the same tendon was demonstrated. Given the floor effect, the measurement error, the clustering of tendons within animals, the timing differences between assessments, and the low power, this absence of detectable correlation should not be generalized into a claim that changes in imaging cannot represent structural pathology; it indicates only that correspondence was not demonstrable in this dataset [50].

## 4. Discussion

In this exploratory controlled pilot study, none of the four mechanistically distinct adjuvant therapies produced a detectable histopathological protective effect against levofloxacin-associated Achilles tendon change. Composite modified Bonar scores did not differ between groups, no individual domain differed and, contrary to the working hypothesis, the levofloxacin-only control did not exhibit the greatest change; the two injectable arms scored numerically higher, while only topical essential oils + ASTM trended lower. The single significant histopathological finding was a within-animal one, and although macroscopic appearance differed between groups, it bore no relationship to the tissue grade of the same tendon. Across the series, the model produced only mild pathology, with a median composite score of 4 of a possible 12 and almost half the tendons scoring 3 or less, which constrains every comparison reported. These preliminary results do not demonstrate a protective effect of PRP, injectable collagen, oral vitamin E + selenium, or topical essential oils + ASTM in this setting, although a pilot of this size can neither establish nor exclude a modest protective effect.

Interpreting these findings requires attention to the molecular sequence through which fluoroquinolones injure the tendon, because that sequence determines when an adjuvant would have to act. The dominant mechanisms converge on the extracellular matrix and are largely front-loaded to the period of drug exposure. Fluoroquinolones chelate divalent cations, particularly Mg2+, disrupting integrin-mediated tenocyte–matrix attachment and the focal-adhesion signaling on which tenocyte survival and matrix synthesis depend [10,43]. They shift the balance between matrix metalloproteinases and their tissue inhibitors toward degradation, so that type I collagen is broken down faster while its synthesis falls [10,17]. In parallel, they generate reactive oxygen species and impair mitochondrial function, depleting glutathione and other antioxidant reserves and driving caspase-dependent tenocyte apoptosis with reduced proliferation and migration [10,17]. More recent syntheses place these events within the wider signaling landscape of tendinopathy, in which redox imbalance, metalloproteinase activation, and impaired tenocyte differentiation reinforce one another [51,52]. Ultrastructural studies in rodents show the resulting phenotype directly: cytoplasmic vacuolation, reduced collagen fibril diameter, and increased interfibrillar spacing still detectable months after exposure [43]. The implication for a prevention study is temporal. If cation chelation, metalloproteinase induction, and the oxidative burst all occur within the seven days of antibiotic exposure, an adjuvant must reach an effective intratendinous concentration inside that same window to alter the outcome; at three months, the tissue reflects not the initial insult but whatever remodeling followed it. Throughout this discussion, such mechanistic considerations are hypotheses drawn from the literature. No molecular, biochemical or biomechanical endpoint was measured in this study; these considerations serve to frame the interpretation of the findings rather than to explain them.

The most important interpretive constraint is that the model itself produced only mild tendinopathy. The control group, which received levofloxacin without any adjuvant, had a median composite score of only 3 of a possible 12. Moreover, because no healthy, levofloxacin-free control group was included, the low scores observed cannot be attributed with certainty to levofloxacin at all. Normal variation in mature rabbit tendons, age-related change, background findings, and processing artefacts could each have contribute, and the causal step from levofloxacin exposure to the observed mild lesions remains undemonstrated in this experiment. For this reason, the tendons studied here are described as levofloxacin-exposed rather than as exhibiting levofloxacin-induced tendinopathy. A robust demonstration of protection requires a control group with substantial, reproducible pathology against which attenuation can be measured. The limited pathology observed here may reflect an insufficient cumulative fluoroquinolone insult at 100 µg/kg/day for seven days in a skeletally mature rabbit, partial structural recovery over the three-month interval between exposure and harvest, or species-specific tendon resilience. This floor effect substantially limited the power to detect any protective signal and is the most likely explanation for the uniformly low scores.

The observation that the two injectable arms trended toward higher rather than lower scores merits caution rather than over-interpretation. A single percutaneous peritendinous injection introduces mechanical and inflammatory stimuli independent of the injected substance, and the higher frequency of peritendinous adhesions and the slightly higher paratendinous-fibrosis and cellularity scores in these groups are compatible with a local needle- or volume-related reaction. Procedural and mechanical components are integral to invasive tendon interventions, which is why such interventions require detailed procedural reporting and carefully matched comparators [53], and clinical experience with high-volume injections in Achilles tendinopathy likewise indicates that the injected volume and the injection procedure can themselves modify tendon outcomes independently of the injected agent [54]. This is consistent with the heterogeneous and often modest effects of PRP reported in established Achilles tendinopathy and suggests that a single prophylactic infiltration is, at best, not protective in this model.

There is also a mechanistic reason why platelet-rich plasma might fail, or even prove counterproductive, when given prophylactically. PRP delivers a bolus of platelet-derived growth factor, transforming growth factor-β, vascular endothelial growth factor, basic fibroblast growth factor, and insulin-like growth factor-1 [19,20]. These are proliferative and profibrotic signals; in particular, TGF-β drives fibroblast activation, type III collagen deposition, and the disorganized matrix that characterizes the disrepair stage of the Cook–Purdam continuum [7,9]. Applied to a tendon that has not yet been injured, such a stimulus may push resident tenocytes toward a reactive, matrix-synthetic phenotype rather than protect them from the oxidative and metalloproteinase-mediated insult that follows. The higher cellularity and morphology grades recorded in the injectable arms are at least compatible with that interpretation, and they argue that the timing of a biological adjuvant relative to the injury may matter as much as its composition.

This impression is reinforced, with caution, by the only comparison in this pilot to reach nominal significance. Within the seven animals that received injectable collagen in one limb and essential oils with augmented soft-tissue mobilization in the other, the collagen-treated tendon scored higher in six of seven pairs (median difference +3.0 points, *p* = 0.031). Because this analysis focused on effects that were internal to each animal, it was not confounded by between-animal variation, which is the principal weakness of the group-level comparison. It nevertheless requires careful interpretation; it compares two active interventions rather than either against the untreated control, it rests on only seven pairs, and it does not survive correction for the two paired comparisons performed (adjusted *p* = 0.062). It should therefore be read as a hypothesis-generating signal, interpretable only against future vehicle-, needle-, and excipient-matched controls, rather than as evidence that collagen itself is harmful or biologically active. The direction is consistent across the three intratendinous domains and absent for paratendinous fibrosis, which is compatible with, but does not establish, a response to the injected material itself; in the absence of a saline, excipient-matched, or needle-only comparator, injection-volume, local anesthetic and mechanical effects cannot be excluded. Confirmation would require a dedicated, adequately powered comparison against both an untreated and a vehicle-injected control; the present estimate of effect size and variability is offered to support the design of exactly such a study.

A molecular reading of this result is instructive. Injectable type I collagen behaves as a mechanical scaffold rather than as a pharmacological agent. Cultured human tenocytes expose to it increased type I collagen synthesis, maturation, and secretion, upregulate the tissue inhibitor of metalloproteinases TIMP-1, and show enhanced focal adhesion and migration through mechanotransduction-related pathways [26,27]. Those are precisely the responses one would wish to invoke against fluoroquinolone-induced matrix loss, since they oppose both arms of the metalloproteinase–inhibitor imbalance described above. The difficulty is one of context. An anabolic, matrix-synthetic stimulus delivered into a peritendinous space that has not yet sustained appreciable damage may generate a matrix response that is unnecessary rather than reparative, and the same upregulation of collagen synthesis that is protective in a degenerate tendon may register histologically as hypercellularity and architectural disorder in a near-normal one. 

The essential oils + ASTM group had the lowest median score, which is mechanistically plausible given the anti-inflammatory properties of the terpene constituents and the pro-remodeling effects attributed to soft-tissue mobilization. However, this difference was small, non-significant, and based on only seven tendons, and it should be regarded as hypothesis-generating rather than evidence of efficacy. It must also be weighed against the macroscopic findings. Peritendinous adhesions were recorded in five of the seven tendons receiving this intervention (Section 3.7); thus, the lowest histopathological score coexisted with the highest frequency of a potentially unfavorable local tissue response. Because the intervention was a compound one including five botanical oils, a vehicle, daily manual restraint, and daily mobilization, with no vehicle-only, oil-only, mobilization-only or handling-matched comparator, neither the lower score nor the adhesions can be attributed to any single component, and the two observations should be read together rather than selectively.

The molecular rationale for this combination is nevertheless coherent and worth stating, because it defines what a properly powered trial would be testing. The terpene constituents of the blend inhibit 5-lipoxygenase and scavenge free radicals, limiting eicosanoid-mediated inflammatory signaling and lipid peroxidation at the site of application [34,35], while augmented soft-tissue mobilization supplies a mechanotransductive stimulus that activates fibroblasts and increases fibronectin and type I collagen synthesis through integrin and focal-adhesion signaling [9,32,33]. In principle, the two act at complementary points of the same pathology: a local antioxidant and anti-inflammatory effect directed at the drug-induced oxidative burst, and a mechanical stimulus favoring aligned rather than disorganized matrix remodeling. Of the four interventions, this was the only one delivered repeatedly, over 21 days, and therefore, the only one plausibly present throughout the at-risk window identified above.

A further observation concerns the relationship between the three assessment modalities. Macroscopic appearance differed significantly between groups, with peritendinous adhesions concentrated in the group that received 21 days of repeated manual mobilization and largely absent from the systemically treated group. Yet, neither macroscopic appearance nor ultrasonographic abnormality predicted the histopathological grade of the same tendon. This dissociation matters beyond the present study, because ultrasonography is frequently used as a surrogate structural endpoint in both preclinical and clinical tendon research. In this series, it identified abnormality in half the tendons while showing no correspondence with the histology of those same specimens. This should be read as an absence of demonstrated correspondence rather than as evidence that imaging is an unreliable surrogate; structural sonographic changes do not map directly onto symptoms, function, or histopathological severity, may be present in asymptomatic tendons, and are sensitive to the anatomical site, transducer position, tendon loading, acquisition plane, and examiner reliability [50]. Comparable dissociations between macroscopic or functional restriction and tissue-level properties have been reported in other small-animal musculoskeletal models [55].

The excess of peritendinous adhesions in the essential oils + ASTM group is itself consistent with a mechanotransductive mechanism. Repeated shear loading of the paratendon upregulates fibronectin and collagen deposition by paratendinous fibroblasts [32,33]; where the gliding surface is intact, that same anabolic response manifests macroscopically as adhesion rather than as useful remodeling. The two modalities also interrogate different molecular substrates, which is why their dissociation is less paradoxical than it first appears. B-mode echogenicity is governed largely by tissue water and proteoglycan content and by the regularity of the collagen fibril lattice, so that it is sensitive to the hydration and proteoglycan changes of the reactive stage [7,9], whereas the Bonar domains scored here capture cellular phenotype, vascular density, and peritendinous fibrosis. Prospective imaging data support this caution; sonographic abnormality in asymptomatic tendons predicts future symptoms only modestly [56]. Imaging and histology may therefore diverge precisely when the dominant abnormality is early and matrix-hydration-based rather than cellular.

The quantitative ultrasonographic data reinforce this point rather than resolving it. Tendon thickness was measured in every animal at baseline and at day 14, yet neither the baseline value nor the change over that interval showed any relationship with the histopathological grade of the same tendon three months later. Ultrasonography in this model therefore detected change; half the tendons were abnormal on qualitative assessment and thickness shifted measurably in one group, without that change corresponding to what was subsequently found in the tissue. Two explanations are plausible and cannot be separated here. The imaging may capture transient peritendinous and fluid changes that resolve before the histological endpoint, or the mild histopathology achieved may simply lie below the threshold at which imaging and tissue findings converge. Either way, these pilot data could not establish a correspondence between the recorded imaging findings and the histological measures; given the largely missing three-month quantitative thickness data, the restricted histological variability and the different assessment timepoints, they do not demonstrate that ultrasonography is an unreliable surrogate, and the question should be tested directly, under a standardized measurement protocol, rather than presumed in either direction.

The one quantitative between-group difference concerned the vitamin E + selenium group, in which tendon thickness increased by a median of 14.1% by day 14 while every other group was unchanged or thinner. This finding requires more caution than its *p*-value suggests. Because vitamin E + selenium was administered to a separate cohort of animals, covariate adjustment for baseline thickness cannot exclude cohort-level confounding, and the observed increase should not be interpreted as a biological effect in the absence of a balanced, independently randomized design. The tendons in that group were already thinner than the others at baseline, before any intervention, and baseline thickness predicted subsequent change across the whole series; so, regression to the mean is an obvious candidate explanation. Adjusting for baseline by analysis of covariance did not abolish the group effect, which argues that regression to the mean is not the whole story; but because the vitamin E + selenium animals were a separate cohort of seven rabbits rather than contralateral limbs of shared animals, a group difference cannot be distinguished from a cohort difference. Covariate-adjusted comparisons of this kind are an established approach for separating supplementation effects from baseline musculoskeletal variability in rodent studies [57], but no adjustment can compensate for a design in which treatment group and animal cohort coincide. In an exploratory pilot with unbalanced baseline characteristics the observation is best regarded as hypothesis-generating. If antioxidant supplementation genuinely produces early tendon swelling, that would be biologically interesting given the reactive stage of the Cook–Purdam continuum, but it would need to be tested prospectively with balanced baseline thickness and a predefined imaging endpoint.

The antioxidant rationale for this arm is the most specific of the four at a molecular level, which makes its failure to alter histopathology the most informative of the negative results. α-Tocopherol terminates lipid peroxidation chain reactions within cell membranes and preserves membrane integrity under oxidative load [28,29], while selenium is incorporated as selenocysteine into the glutathione peroxidases and thioredoxin reductases that constitute the principal enzymatic defence against peroxides, so that selenium status sets the effective ceiling on glutathione peroxidase activity [30]. Selenium supplementation acts on tendon cells directly as well as systemically. Selenium delivered to tendon-derived stem/progenitor cells under peroxide stress reduces oxidative injury, inflammation, and apoptosis and restores tenocyte marker expression through Sirt1- and Nrf2-dependent signaling [58], and hypoxia-inducible factor pathways are increasingly recognized as a further node linking redox state to tendon degeneration [59]. The two are synergistic because they act at different points of the same pathway, enzymatic reduction of peroxides on the one hand and non-enzymatic chain termination on the other [28,31]. Given that fluoroquinolone tenotoxicity is substantially oxidative, this arm targeted the mechanism most directly. Two explanations for its failure deserve consideration. A systemic dose may not achieve an intratendinous concentration sufficient to counter a local oxidative burst in a tissue that is by design poorly vascularized, or the mild insult achieved may not have generated enough oxidative stress for an antioxidant effect to be demonstrable. An early increase in tendon thickness without corresponding histological change would also be consistent with a reactive-stage response, in which increased proteoglycan synthesis by activated tenocytes binds water and raises tendon volume without disrupting collagen [7,9].

These histopathological findings contrast with the clinical signal reported by our group in a 2025 randomized pilot, in which concomitant vitamin E and selenium supplementation during levofloxacin therapy was associated with lower pain and better functional and ultrasonographic outcomes [16]. This divergence may reflect differences in species, endpoint (patient-reported and ultrasonographic outcomes versus histology), exposure intensity, and the mild pathology achieved in the present model. Equally, the earlier clinical signal cannot be taken to strengthen the biological interpretation of the present animal data: the populations, exposures, and outcomes are fundamentally different, and neither study validates the other. It underscores that clinical and histopathological endpoints are not interchangeable and that a negative histological result in a low-severity animal model does not refute a clinical benefit. Similar interpretive difficulties have been described for other drug-associated tendon disorders, including statin-associated tendinopathy, in which overlapping mechanisms such as extracellular-matrix dysregulation and altered matrix metalloproteinase activity coexist with considerable uncertainty of causal inference in heterogeneous clinical evidence [60].

Two measurement observations have implications for the design of future studies. First, Achilles tendon thickness in rabbits varies markedly along its length, being roughly 3.5 mm greater 35 mm from the calcaneal insertion than at 20 mm, so that the level of measurement must be standardized and reported if thickness is to serve as an endpoint. The same problem is documented in human patients in whom ultrasonographic thickness is measurably less reliable at the insertion than at the midportion [61]. Standardizations of the anatomical site, transducer position, limb loading, acquisition plane, and examiner reliability are accordingly prerequisites for thickness-based endpoints [50]. Second, ultrasonography performed post mortem underestimated direct caliper measurement of individual specimens by a mean of 0.65 mm, with limits of agreement spanning almost 2.5 mm. Imaging and direct measurement are therefore not interchangeable at the scale of the differences being sought in this model, which is a further reason to treat small imaging changes with caution.

The clinical translatability of prophylactic invasive procedures also deserves explicit comment. Fluoroquinolone-associated tendon injury is uncommon relative to the number of prescriptions, and current prescribing practice already emphasizes avoiding systemic fluoroquinolones when safer alternatives are suitable [15]. Routine injection of an asymptomatic tendon with PRP or collagen would impose cost, procedural risk, and local tissue trauma on many patients who would never have developed tendinopathy, and the risk–benefit rationale for invasive prophylaxis therefore differs fundamentally from that of a low-risk oral or topical intervention; any future development of injectable prophylaxis would require both validated risk prediction to select recipients and a far stronger demonstration of efficacy. More broadly, treatment effects in tendon disorders depend strongly on anatomical site, pathological mechanism, disease stage, comparator, and outcome, and evidence generated in chronic symptomatic tendinopathy or tenosynovitis cannot be extrapolated automatically to the prevention of acute drug-induced tendon toxicity [62]; this reinforces the recommendation below that future experiments should evaluate fewer interventions against appropriate sham or vehicle controls rather than several dissimilar therapeutic packages simultaneously.

Several limitations qualify these conclusions. The most fundamental is that this was an exploratory pilot study; it was not powered for confirmatory inference, no minimum detectable effect was specified, and the number of animals was set by practical and 3Rs considerations rather than by a formal calculation. Every comparison reported should therefore be read as an estimate of direction and variability rather than as a test of efficacy. First, and most importantly, the mild pathology in the control group produced a floor effect. Second, the sample was small, with only seven tendons in each locally treated group, giving limited power to detect modest effects; no formal a priori power analysis was performed, although the sensitivity analysis reported in Section 2.4 quantifies the minimum effect sizes detectable with the achieved sample. Third, the contralateral-limb design means the tendons were not fully independent. Oil- and collagen-treated tendons came from the same animals, as did the PRP and control tendons, and the systemic vitamin E + selenium arm contributed two tendons per animal, whereas the between-group tests assumed independence. Moreover, because the locally treated tendons were consistently assigned to the same limb within each cohort (collagen and PRP to the left, their comparators to the right), treatment was partially confounded with limb side. Fourth, scoring relied on a study-specific modified Bonar composite that omitted the classic ground-substance and collagen-organization domains; because extracellular-matrix disruption is a hallmark of fluoroquinolone toxicity, this may have under-captured relevant change, and it limits comparability with other studies. Fifth, only a single reconciled consensus score was available per tendon, meaning that inter-observer reliability could not be quantified; likewise, all ultrasonographic scans were performed by a single experienced examiner and no intra-rater test-retest reliability was assessed, and measurement error therefore cannot be bounded at the scale of the observed thickness differences. Finally, this study used a single three-month histological timepoint, which may reflect remodeling or partial recovery rather than the early injury that prophylaxis was intended to prevent, and qualitative ultrasonography was employed without molecular or biochemical endpoints such as matrix metalloproteinase activity, collagen typing, or oxidative-stress markers. In interpretive terms, the present results therefore constitute absence of evidence of benefit rather than evidence against benefit and, with the exception of the local macroscopic findings discussed above, they do not constitute evidence of harm. Additional limitations deserve mention. The systemic vitamin E + selenium condition comprised seven experimental units (animals) rather than fourteen tendons; the group-specific fixation durations and processing batches confounded treatment with processing batch in the between-group histological comparisons; no sham- or vehicle-injection comparator and no levofloxacin-free healthy control group were included, meaning that procedural effects cannot be separated from product effects and drug-induced pathology cannot be separated from background change; the four interventions differed in route, invasiveness, dose frequency, and duration (single injections versus 14 or 21 days of repeated administration), and they therefore cannot be interpreted as directly comparable treatment strategies; the levofloxacin exposure achieved is unverified, as an intravenous formulation was administered orally without pharmacokinetic confirmation; and, because no gait, limb-loading, or pain-behavior assessment was performed, biological carryover between the two limbs of an animal—through altered loading after a painful injection or repeated mobilization of the contralateral side—cannot be excluded, which means that the independence of local tendon responses within an animal is an assumption rather than an observation.

Three further limitations follow from the additional data. Baseline tendon thickness was not balanced across groups, which was not appreciated at the design stage and which complicates interpretation of the only quantitative between-group difference. Quantitative thickness was obtained at the three-month examination in only 3 of 42 tendons; therefore, the longitudinal imaging analysis is confined to the first 14 days and cannot speak to the endpoint at which histopathology was assessed. Finally, the ex vivo caliper measurements are not comparable across all five groups, because the plantaris tendon was retained en bloc in four groups and excluded in the vitamin E + selenium group; this makes those measurements uninformative for between-group comparison and they are reported for completeness only.

Future work should first establish a rabbit model that reliably reproduces moderate-to-severe fluoroquinolone tendinopathy through higher or more prolonged dosing, adjunctive risk factors, or earlier harvest, before adjuvant efficacy can be meaningfully tested, following the model-development approach applied to mechanically induced Achilles tendinopathy, in which candidate induction protocols are compared head-to-head using the Bonar score as the quantitative indicator [63]. Subsequent studies will benefit from adequately powered independent groups with randomized limb allocation, sham- and vehicle-injection comparators, a levofloxacin-free control group, early histological assessment within the expected injury window, repeated rather than single adjuvant dosing, specimen processing randomized across batches, the full Bonar composite supplemented by matrix and oxidative-stress endpoints, and inter-observer reliability quantified from retained independent scores [49].

## 5. Conclusions

Within the limits of this exploratory controlled pilot study, no statistically detectable difference in the study-specific four-domain composite score was identified among the five treatment conditions—local PRP, injectable porcine collagen, oral vitamin E + selenium, topical essential oils delivered by augmented soft-tissue mobilization, and a levofloxacin-only control—in levofloxacin-exposed Achilles tendons. The model produced only mild pathology, and the two injectable arms trended toward more rather than less change; in the within-animal comparison that the contralateral-limb design permits, tendons receiving injectable collagen scored higher than the paired essential-oil tendons. This was a nominal signal that did not survive correction for multiple comparisons and could not be separated from needle-, volume-, vehicle- or side-related effects. Quantitative ultrasonography detected an early increase in tendon thickness confined to the vitamin E + selenium group, but this was confounded by a baseline imbalance and did not correspond to any histopathological difference. Indeed, no correspondence between ultrasonographic or macroscopic findings and the tissue-level grade of the same tendon could be demonstrated, although the largely missing three-month quantitative measurements and the narrow range of histological change limit what this pilot can conclude about imaging as a structural surrogate. Because the model induction was mild and outcome sensitivity was limited, these preliminary findings remain inconclusive regarding the prophylactic use of these adjuvants in fluoroquinolone-associated tendinopathy; as an exploratory pilot, this study is intended to inform the design of an adequately powered confirmatory trial rather than to establish or exclude efficacy. Such a trial will require a model producing more substantial and reproducible pathology validated against a levofloxacin-free control, randomized limb allocation, balanced baseline characteristics, standardized measurement levels, and both vehicle-injected and untreated comparators.

## Figures and Tables

**Table 1:** Botanical identity, source, supplier, and manufacturer-reported specifications of the five essential oils and the vehicle used in the topical anti-inflammatory formulation. INCI, International Nomenclature of Cosmetic Ingredients; HEBBD, botanically and biochemically defined essential oil.

Component	Botanical Source/INCI Name	Plant Part and Origin	Supplier	Physicochemical Specification	Principal Constituents (Manufacturer Data)
Balsam fir essential oil	*Abies balsamea* (L.) Mill.; INCI *Abies balsamea* Needle Oil (code M-1382)	Needles and twigs; Canada	Ellemental SRL, Oradea, Romania	Relative density 0.865–0.885; refractive index 1.472–1.480 (20 °C); COSMOS organic certified	β-pinene 28–38%; α-pinene 9–19%; Δ^3^-carene 1–17%; bornyl acetate 5–12%; d-limonene 5–10%
Lemon eucalyptus essential oil	*Eucalyptus citriodora* Hook. (syn. Corymbia citriodora); INCI *Eucalyptus citriodora* oil (code M-1326)	Leaves and twigs	Ellemental SRL, Oradea, Romania	Relative density 0.860–0.885; refractive index 1.445–1.460; optical rotation −5° to +5° (20 °C); clear colorless to light-yellow liquid	Not specified by the manufacturer
Katrafay essential oil	Cedrelopsis grevei Baill.; INCI Cedrelopsis grevei Oil	Bark; Madagascar	MALA—Madagascar Aromatic, Ambanja, Madagascar	Relative density 0.915–0.945; refractive index 1.480–1.510 (20 °C); optical rotation −20° to +15°; flash point > 65 °C; certified organic	Ishwarane 15–48%; α-copaene 5–12%; β-sesquiphellandrene 8–12%; α-curcumene 3–11%; β-bisabolene 3–7%; E,E-α-farnesene 2.5–8.5%; β-selinene 1–10%; α-selinene 1–10%; δ-cadinene 1–10%; geranial 0.5–3.5%; geraniol 0.1–3.5%
Italian immortelle essential oil	*Helichrysum italicum* G. Don; INCI *Helichrysum italicum* Flower Oil	Flowering tops; cultivated in Italy	Biolandes SAS, Le Sen, France	Relative density 0.880–0.920; refractive index 1.460–1.475; optical rotation −20° to +10° (20 °C); certified organic	Neryl acetate 3–42%; γ-curcumene 4–20%; α-pinene 0–20%; italidiones 1–15%
Mastic tree essential oil	*Pistacia lentiscus* L.; INCI *Pistacia lentiscus* Leaf Oil	Twigs; organically cultivated, Morocco	Florame Laboratoires, Saint-Rémy-de-Provence, France	Relative density 0.850–0.875; refractive index 1.465–1.485; optical rotation −13° to +4° (20 °C); HEBBD-defined	Not specified by the manufacturer
Vehicle	Fitalite™ hydrophilic oil-in-water gel-cream base	–	Fagron UK Ltd., Newcastle upon Tyne, UK	pH 3.5–5.5; light-yellow opaque gel-cream; free of fragrance, dyes, parabens, mineral oils, sodium lauryl sulfate, propylene glycol, and 1,4-dioxane; stored below 25 °C	Purified water; Carthamus tinctorius oleosomes; polyacrylate-13; polyisobutene; sodium carbomer; polysorbate 20; tocopheryl acetate; benzoic acid; sorbic acid

**Table 2:** Composite modified Bonar histopathological score (range 0–12) by experimental group at three months. Kruskal–Wallis H = 3.663, df = 4, *p* = 0.45. IQR, interquartile range; ASTM, augmented soft-tissue mobilization; PRP, platelet-rich plasma; SD, standard deviation.

Group	n	Median [IQR]	Mean ± SD	Min	Max	Tendons with ≥1 Domain ≥ Grade 2
Essential oils + ASTM	7	2.00 [1.50–3.50]	2.43 ± 1.72	0	5	3/7 (43%)
levofloxacin-only control	7	3.00 [2.00–4.50]	3.14 ± 2.04	0	6	5/7 (71%)
Vitamin E + selenium	14	3.50 [1.00–5.75]	3.57 ± 2.98	0	8	7/14 (50%)
PRP	7	4.00 [2.00–6.50]	4.29 ± 2.75	1	8	6/7 (86%)
Injectable collagen	7	4.00 [4.00–6.50]	4.86 ± 2.34	1	8	6/7 (86%)

**Table 3:** Individual modified Bonar domain scores by group, expressed as median [interquartile range]. Each domain is graded 0 (normal) to 3 (markedly abnormal). H and *p* are from the Kruskal–Wallis test across the five groups (df = 4).

Domain	Essential Oils + ASTM	levofloxacin-Only Control	Vitamin E + Selenium	PRP	Injectable Collagen	H	*p*
Tenocyte morphology	0.00 [0.00–0.50]	1.00 [0.00–1.00]	1.00 [0.00–1.00]	0.00 [0.00–2.00]	1.00 [0.50–2.50]	2.650	0.62
Cellularity	0.00 [0.00–0.50]	0.00 [0.00–1.00]	1.00 [0.00–1.75]	1.00 [0.00–2.00]	1.00 [0.50–2.00]	4.498	0.34
Vascularity	1.00 [0.00–1.00]	2.00 [1.00–2.00]	0.50 [0.00–1.00]	1.00 [0.00–1.50]	2.00 [1.00–2.00]	5.178	0.27
Paratendinous fibrosis	1.00 [0.00–1.50]	0.00 [0.00–0.50]	0.50 [0.00–2.00]	1.00 [0.50–2.00]	0.00 [0.00–1.50]	3.549	0.47

**Table 4:** Within-animal comparisons of the composite modified Bonar score, using the contralateral-limb design.

Comparison (Within Animal)	Pairs	Median Difference	Higher in	Wilcoxon *p*	Bonferroni-Adjusted *p*
Injectable collagen vs. essential oils + ASTM	7	+3.0	6/7 collagen (1 tie)	0.031	0.062
PRP vs. levofloxacin-only control	7	+2.0	4/7 PRP	0.63	1.00
Vitamin E + selenium, left vs. right limb	7	−2.0	3/7 left	0.53	–

Positive differences indicate a higher (worse) composite modified Bonar score in the first-named group.

**Table 5:** Macroscopic findings recorded at dissection, by experimental group.

Group	Adhesions	Hyperaemia	Any Macroscopic Finding
Essential oils + ASTM	5/7 (71%)	0/7 (0%)	5/7 (71%)
Levofloxacin-only control	2/7 (29%)	3/7 (43%)	5/7 (71%)
Vitamin E + selenium	2/14 (14%)	0/14 (0%)	2/14 (14%)
PRP	1/7 (14%)	1/7 (14%)	2/7 (29%)
Injectable collagen	3/7 (43%)	1/7 (14%)	4/7 (57%)
All tendons	13/42 (31%)	5/42 (12%)	18/42 (43%)

Permutation test for any macroscopic finding across the five groups, *p* = 0.031. Tendons without a recorded abnormality were classified as showing no macroscopic change.

**Table 6:** In vivo ultrasonographic Achilles tendon thickness at baseline and day 14, by experimental group. The change column summarizes the median of the within-animal paired differences, which need not equal the arithmetic difference between the two marginal medians.

Group	Baseline (mm)	Day 14 (mm)	Change (mm)	Change (%)	Within-Group *p*
Essential oils + ASTM	6.00 [5.80–6.10]	5.60 [5.40–6.10]	−0.30 [−0.65, −0.10]	−5.3	0.38
levofloxacin-only control	6.20 [6.00–6.45]	5.80 [5.75–6.00]	−0.20 [−0.45, −0.05]	−3.3	0.27
Vitamin E + selenium	5.20 [4.70–5.62]	5.65 [5.30–6.82]	+0.70 [+0.13, +1.42]	+14.1	0.007
PRP	6.00 [5.75–6.05]	5.40 [5.05–5.75]	−0.80 [−1.00, −0.20]	−12.5	0.078
Injectable collagen	6.10 [5.80–6.25]	6.00 [5.55–6.45]	−0.10 [−0.25, +0.30]	−1.6	1.00

Values are median [interquartile range]. Between-group comparison of absolute change, Kruskal–Wallis *p* = 0.003; and relative change, *p* = 0.003. Analysis of covariance of day-14 thickness adjusted for baseline, group effect F(4,36) = 4.35, *p* = 0.006.

**Table 7:** Ex vivo specimen measurements by group.

Measurement	Essential Oils + ASTM	levofloxacin-Only Control	Vitamin E + Selenium	PRP	Injectable Collagen
Post-mortem US, 20 mm	4.20 [4.10–4.90]	4.10 [3.85–4.35]	3.70 [3.35–3.77]	4.30 [4.10–4.60]	4.60 [4.35–4.80]
Caliper, 20 mm	5.00 [4.70–5.60]	4.50 [4.40–5.20]	3.95 [3.75–4.38]	5.00 [4.85–5.20]	5.30 [5.00–5.58]
Caliper, 35 mm	9.00 [8.40–9.35]	8.90 [8.55–9.20]	6.00 [6.00–6.30]	8.60 [8.35–9.15]	8.70 [8.25–9.75]
Proximal–distal difference	4.10	4.10	2.00	3.50	3.90

Values are median [interquartile range] in millimeters. In the four groups other than vitamin E + selenium, the plantaris tendon was included in the harvested specimen; caliper measurements are therefore not comparable across all five groups. No between-group inferential comparison is presented, because the apparent group differences are determined by specimen composition rather than by treatment (Section 3.10).

## Data Availability

The original contributions presented in this study are included in the article/Supplementary Material. Further inquiries can be directed to the corresponding authors.

## Supplementary Materials

The following supporting information can be downloaded at https://www.mdpi.com/article/10.3390/jcm15176596/s1, Table S1: Raw data; the completed ARRIVE 2.0 checklist; and the analysis code reproducing all reported statistics are provided in a single supplementary PDF file, in this order.
