# Supplementary material for: Adjuvant Interventions in Levofloxacin-Exposed Rabbit Achilles Tendons: An Exploratory Controlled Pilot Study with Histopathological and Ultrasonographic Endpoints
Source: J Clin Med. 2026 Aug 26;15(17):6596. doi: 10.3390/jcm15176596 (PMC13565932; doi:10.3390/jcm15176596)
Supplement: Supplementary file 1 [file jcm-15-06596-s001.zip › jcm-4487171-supplementary.pdf]

Supplementary Materials

Table S1. Complete raw dataset for all 42 Achilles tendons from 21 rabbits. For each tendon the table gives the animal identifier, experimental group, limb side and body weight; the four scored modified Bonar domains (each graded 0–3) and the composite score (0–12); in vivo ultrasonographic tendon thickness at baseline (day 1), at day 14 and at three months, with the absolute and relative change over the first interval; post-mortem ultrasonographic thickness and direct caliper measurements of the harvested specimen at 20 mm and 35 mm from the calcaneal insertion; and the qualitative macroscopic and ultrasonographic findings recorded for that tendon. Blank cells in the descriptor columns denote no abnormality detected; blank cells in the three-month thickness column denote a measurement that was not obtained.

Tendon ID	Group	Side	Weight (kg)	Mor	Cel	Vas	Fib	Total	US d1	US d14	US 3 mo	Δ mm	Δ %	PM US 20 mm	Cal 20 mm	Cal 35 mm	Macroscopic finding	Ultrasonographic finding
560 DR ulei	Essential oils + ASTM	R	4.23	0	0	2	2	4	6.2	5.4		-0.8	-12.9	4.1	5.0	9.2	aderente la nivelul paratendonului	
563 DR ulei	Essential oils + ASTM	R	4.40	3	1	1	0	5	5.7	5.4		-0.3	-5.3	4.9	5.8	9.5		zone de hipoecogenitate neuniforme la 3 luni
565 DR ulei	Essential oils + ASTM	R	3.93	1	1	0	0	2	6.0	5.4		-0.6	-10.0	4.2	4.0	8.1	aderente la nivelul paratendonului	
566 DR ulei	Essential oils + ASTM	R	4.07	0	0	1	2	3	5.9	5.6		-0.3	-5.1	4.0	4.4	8.7		modificari pe paratendon profund si hiperecogenitate
570 DR ulei	Essential oils + ASTM	R	4.74	0	0	0	1	1	6.1	7.2		+1.1	+18.0	4.9	6.9	8.0	aderente paratendon la nivelul insertiei calcaneene	aspect ingrosat la 3 luni
574 DR ulei	Essential oils + ASTM	R	3.91	0	0	0	0	0	6.1	5.4		-0.7	-11.5	4.1	5.4	9.0	aderente la nivelul paratendonului	
794 DR ulei	Essential oils + ASTM	R	3.98	0	0	1	1	2	5.5	5.6		+0.1	+1.8	4.9	5.0	9.5	aderente la nivelul paratendonului	
551 D CONTROL	Untreated negative control	R	4.05	1	0	2	1	4	6.3	5.9		-0.4	-6.3	4.4	4.4	8.6	aderente la nivelul paratendonului	Hiperecogenitate paratendon, deorganizare fibrilara la 3 luni
554 D CONTROL	Untreated negative control	R	4.12	3	2	1	0	6	6.2	5.7		-0.5	-8.1	4.1	6.1	9.5	hiperemie la nivelul paratendonului la insertia pe calcaneu	deorganizare fibrilara la 3 luni
559 D CONTROL	Untreated negative control	R	3.40	0	1	1	0	2	6.0	5.8		-0.2	-3.3	3.9	4.4	8.5	hiperemie la nivelul paratendonului	Hiperecogenitate paratendon, deorganizare fibrilara la 3 luni
572 D CONTROL	Untreated negative control	R	4.00	1	1	2	1	5	6.0	5.8		-0.2	-3.3	4.3	4.4	8.9	hiperemie la nivelul paratendonului	lichid peritendon, deorganizare fibrilara la 14 zile
777 D CONTROL	Untreated negative control	R	4.09	0	0	0	0	0	6.6	6.7		+0.1	+1.5	4.7	5.3	8.9		
788 D CONTROL	Untreated negative control	R	4.34	1	0	2	0	3	5.4	6.1		+0.7	+13.0	3.8	4.5	6.8	aderente la nivelul paratendonului	
795 D CONTROL	Untreated negative control	R	3.89	0	0	2	0	2	6.6	5.7		-0.9	-13.6	3.4	5.1	10.3		
552 D vit e	Vitamin E + selenium	R	4.19	2	2	2	2	8	6.2	7.0		+0.8	+12.9	3.8	3.7	6.0		
552 S vit e	Vitamin E + selenium	L	4.19	1	1	0	0	2	5.3	6.9		+1.6	+30.2	3.7	3.5	5.3		
553 D vit e	Vitamin E + selenium	R	3.68	1	3	3	1	8	5.7	5.7		+0.0	+0.0	4.2	4.4	6.3		
553 S vit e	Vitamin E + selenium	L	3.68	0	0	0	0	0	5.9	6.4	5.8	+0.5	+8.5	3.3	4.4	6.1		hipoecogenitate si peritendon usor modificat la 14 zile si cu zone de discontinuitate tendinoasa si aspect fusiform ingrosat
556 D vit e	Vitamin E + selenium	R	3.94	0	0	0	0	0	5.3	4.8		-0.5	-9.4	3.5	4.1	6.6		
556 S vit e	Vitamin E + selenium	L	3.94	1	2	1	0	4	4.2	4.9		+0.7	+16.7	3.7	4.9	6.0	aderente la nivelul paratendonului	leziuni focale la nivelul tendonului la 14 zile cu zone de discontinuitate
567 D vit e	Vitamin E + selenium	R	4.30	0	0	0	1	1	5.0	6.6		+1.6	+32.0	3.7	3.9	6.0		hiperecogenitate fibrilara a tendonului ahilian la 14 zile
567 S vit e	Vitamin E + selenium	L	4.30	3	0	0	2	5	4.6	5.3		+0.7	+15.2	4.0	3.7	6.0		
573 D vit e	Vitamin E + selenium	R	4.50	1	1	1	2	5	5.4	7.4		+2.0	+37.0	4.8	3.9	5.6		
573 S vit e	Vitamin E + selenium	L	4.50	0	0	0	0	0	4.4	7.0		+2.6	+59.1	3.7	3.3	5.0		dezorganizare si aspect neomogen la nivelul fibrelor tendinoase ale tendonului ahilean la 14 zile
779 D vit e	Vitamin E + selenium	R	4.35	1	3	0	2	6	5.7	5.5		-0.2	-3.5	3.0	4.3	7.2	aderente la nivelul paratendonului	aspect neomogen la nivelul tendonului ahilean la 14 zile cu zone de discontinuitate
779 S vit e	Vitamin E + selenium	L	4.35	2	1	2	2	7	5.1	5.3		+0.2	+3.9	3.5	4.4	6.0		aspect ingrosat la 3 luni
784 D vit e	Vitamin E + selenium	R	4.19	0	0	1	0	1	4.5	5.4	6.0	+0.9	+20.0	2.6	3.9	6.8		
784 S vit e	Vitamin E + selenium	L	4.19	1	1	1	0	3	5.1	5.2	5.1	+0.1	+2.0	3.0	4.0	6.3		
551 PRP S	PRP	L	4.05	2	2	1	1	6	6.0	5.0		-1.0	-16.7	4.5	5.4	8.6	aderente la nivelul paratendonului	dezorganizare si aspect neomogen
554 PRP S	PRP	L	4.12	0	0	0	2	2	6.4	5.6		-0.8	-12.5	4.7	5.0	7.8	hiperemie la nivelul paratendonului la insertia pe calcaneu	aspect fusiform fara modificare intratendinoasa la 3 luni
559 PRP S	PRP	L	3.40	0	0	1	0	1	5.7	4.0		-1.7	-29.8	3.7	4.8	8.3		aspect fusiform fara modificare intratendinoasa

572 PRP S	PRP	L	4.00	0	0	0	2	2	6.1	5.1		-1.0	-16.4	4.0	4.8	9.0		
777 PRP S	PRP	L	4.09	2	3	2	1	8	5.6	5.8		+0.2	+3.6	4.2	5.0	8.4		
788 PRP S	PRP	L	4.34	3	2	0	2	7	5.8	6.1		+0.3	+5.2	4.3	4.9	9.4		deorganizare fibrilara la 3 luni
795 PRP S	PRP	L	3.89	0	1	3	0	4	6.0	5.4		-0.6	-10.0	4.8	5.8	9.3		
560 COLAGEN S	Injectable collagen	L	4.23	1	1	2	0	4	6.2	6.0		-0.2	-3.2	4.3	6.2	7.9		zone de hipoecogenitate neuniforme la 3 luni
563 COLAGEN S	Injectable collagen	L	4.40	2	2	2	2	8	6.1	6.0		-0.1	-1.6	4.7	5.3	11.2		
565 COLAGEN S	Injectable collagen	L	3.93	1	1	2	0	4	6.0	6.6		+0.6	+10.0	5.4	5.7	8.6	aderente la nivelul paratendonului	
566 COLAGEN S	Injectable collagen	L	4.07	3	3	1	0	7	6.3	6.3		+0.0	+0.0	4.3	5.0	7.8	aderente la nivelul paratendonului	modificari pe paratendon profund si hiperecogenitate
570 COLAGEN S	Injectable collagen	L	4.74	0	0	2	2	4	5.5	5.2		-0.3	-5.5	4.4	5.0	9.5	hiperemie la nivelul paratendonului	aspect neomogen la nivelul fibrelor tendinoase la 14 zile
574 COLAGEN S	Injectable collagen	L	3.91	0	0	0	1	1	9.5	10.2		+0.7	+7.4	4.6	4.8	8.7	aderente la nivelul paratendonului	dezorganizare si aspect neomogen la nivelul fibrelor tendinoase ale tendonului ahilean la 14 zile, zone de hipoecogenitate neuniforme in structura tendonului la 3 luni
794 COLAGEN S	Injectable collagen	L	3.98	3	2	1	0	6	5.6	5.1		-0.5	-8.9	4.9	5.5	10.0		

Mor, tenocyte morphology; Cel, cellularity; Vas, vascularity; Fib, paratendinous fibrosis; L, left; R, right; US, ultrasonographic thickness (mm); PM, post-mortem; Cal, caliper measurement of the harvested specimen (mm); Δ, change in in vivo thickness between day 1 and day 14. In vivo ultrasonography was performed with an Esaote MyLab system and an SL1543 linear-array probe (3–13 MHz, 55 mm footprint). Four domain entries originally recorded as two-point ranges were resolved by consensus to the higher recorded integer. Caliper measurements in all groups except vitamin E + selenium were obtained from specimens harvested en bloc with the plantaris tendon and are therefore not directly comparable with that group.

ARRIVE 2.0 Checklist

Manuscript jcm-4487171 — Adjuvant Interventions in Levofloxacin-Exposed Rabbit Achilles Tendons: An Exploratory Controlled Pilot Study with Histopathological and Ultrasonographic Endpoints. Item numbering follows ARRIVE 2.0 (Percie du Sert et al., PLoS Biol 2020;18:e3000410); items 1–10 are the "Essential 10", items 11–21 the "Recommended Set". Section numbers refer to the revised manuscript.

Item	Recommendation (ARRIVE 2.0)	Location / status in the revised manuscript
1a,b	Study design: groups, experimental unit	Sections 2.2, 2.6 and 2.18: three-cohort structure with two within-animal paired comparisons and one systemic cohort; experimental unit defined per intervention (tendon within animal block for paired local interventions; animal for the systemic intervention).
2a,b	Sample size: exact numbers, how determined	Sections 2.3, 2.4 and 3.1: 21 rabbits / 42 tendons, no attrition; pragmatic sample size (no a priori power calculation), with post hoc minimum-detectable-effect analysis reported in Section 2.4.
3a,b	Inclusion and exclusion criteria; exclusions reported	Sections 2.3 and 3.1: no formal a priori inclusion/exclusion criteria beyond the animal specification (skeletally mature New Zealand White rabbits, aged 3–4 years) were predefined; no animal or tendon was excluded and there was no attrition.
4a,b	Randomisation: method, sequence generation, allocation concealment	Sections 2.4 and 2.6: sealed opaque envelopes prepared and drawn by the principal investigator were used for the allocation of animals among the cohorts only; assignment of treatments to limb side was fixed by protocol rather than randomized (injected interventions to the left limb, comparators to the right limb), so the treatment–side confound is disclosed as a limitation. No independent allocation concealment (same investigator prepared and drew envelopes).
5	Blinding: who was blinded at each stage	Section 2.4: histopathologists and ultrasonographer blinded, slides coded; intervention personnel not blinded (visibly different procedures).
6a,b	Outcome measures: all assessed outcomes; primary outcome	Section 2.2: primary outcome = four-domain modified Bonar composite (0–12); secondary = domain scores, ultrasonographic thickness; descriptive = macroscopic and qualitative ultrasonographic findings.
7a,b	Statistical methods, incl. software and assumptions	Section 2.18: non-parametric tests, paired analyses, mixed model, ANCOVA, exact CIs, Benjamini–Hochberg correction; experimental-unit caveats; software listed. Full analysis code included in the supplementary PDF.
8a,b	Experimental animals: species, strain, sex, age, weight, source	Section 2.3: male New Zealand White rabbits, aged 3–4 years, 3.40–4.74 kg, Cantacuzino National Institute. Health/pathogen status is not reported in the manuscript.
9a,b,c	Experimental procedures: what, when, where, why	Sections 2.5–2.14: oral levofloxacin by syringe (single daily administration); interventions under mild restraint with local anesthesia (PRP, collagen) or awake (oils + ASTM); general anesthesia for harvest only; ultrasound at midportion (MyLab

Item	Recommendation (ARRIVE 2.0)	Location / status in the revised manuscript
		X5); H&E staining and blinded consensus scoring.
10a,b,c	Results: for each analysis, summary + effect size with CI	Section 3, Tables 2–7: medians with IQR; paired median differences with exact 95.3% CIs (Table 4, Section 3.4); ANCOVA contrast with p; BH-adjusted p-values reported in Sections 3.4, 3.7, 3.9.
11a,b	Abstract: species, key methods, results, conclusion	Abstract: revised to state the allocation structure, experimental units and the inconclusive-for-efficacy framing.
12a,b	Background: scientific context, rationale for animal model	Introduction: fluoroquinolone tenotoxicity, rationale for each adjuvant, and (revised) explicit statement that the induction regimen was not previously validated.
13	Objectives: specific research question	Introduction, final paragraph: exploratory pilot objectives (feasibility, direction and variability of effects, design of a future trial).
14	Ethical statement	Section 2.1 and Institutional Review Board Statement: university, hospital and veterinary authority approvals; Directive 2010/63/EU; Ordinance 42/2004 (Helsinki/GCP removed in revision as inapplicable to animal research).
15	Housing and husbandry	Section 2.3: individual housing, 20 ± 2 °C, 50 ± 10% relative humidity, 12 h light/dark cycle, ad libitum chow and water, free locomotion between procedures. Acclimatization period and environmental enrichment are not reported.
16a,b	Animal care and monitoring; welfare assessments; humane endpoints	Sections 2.3, 2.7, 2.14: daily structured observation record (intake, weight, posture/gait, grooming, pain/distress signs) with predefined thresholds for veterinary reassessment; the approved protocol specifically authorized omission of post-procedural analgesia; no threshold was reached.
17a,b	Interpretation: results in context, limitations	Section 4: floor effect, absence of unexposed control, experimental-unit limits, confounds (side, cohort, processing batch), evidential taxonomy; ten enumerated limitations.
18	Generalisability / translation	Section 4 (revised): clinical-translatability paragraph on invasive prophylaxis; explicit statement that findings are exploratory and hypothesis-generating.
19	Protocol registration	Section 2.2 (revised): the research question, key design features and analysis plan were specified in the protocol approved by the ethics committees and the competent veterinary authority; no additional public registration.
20	Data access	A single supplementary PDF file contains Table S1 (complete raw per-tendon dataset), this checklist, and the analysis code reproducing all reported statistics; see the Supplementary Materials and Data Availability Statements.
21	Declaration of interests	Conflicts of Interest statement: the authors declare no conflicts

Item	Recommendation (ARRIVE 2.0)	Location / status in the revised manuscript
		of interest.

Supplementary analysis code

Self-contained Python script reproducing every inferential statistic reported in the manuscript from the raw per-tendon dataset (Table S1). Requirements: Python >= 3.10, numpy, scipy >= 1.10, statsmodels >= 0.14.

```
# -*- coding: utf-8 -*-
"""
Supplementary analysis code
===========================
Manuscript: "Adjuvant Interventions in Levofloxacin-Exposed Rabbit
Achilles
Tendons: An Exploratory Controlled Pilot Study with Histopathological
and
Ultrasonographic Endpoints" (jcm-4487171, revised).

Self-contained script reproducing every inferential statistic reported
in the
revised manuscript from the raw per-tendon dataset (Supplementary
Table S1).
Requirements: Python >= 3.10, numpy, scipy >= 1.10, statsmodels >=
0.14.
Run:  python Supplementary_analysis_code.py

Note: the manuscript reports some Wilcoxon p-values from the normal
approximation; this script prefers exact methods where feasible, so
small
differences (e.g., 0.63 vs 0.69 for the PRP paired comparison, 0.007
vs 0.004
for the vitamin E within-group change) reflect the method variant, not
the
data. The permutation p-value is Monte Carlo (B = 20,000, seeded) and
varies
by ~0.005 around the reported 0.031.

Sections:
  1. Raw data (transcribed from Supplementary Table S1)
  2. Descriptive summaries (Tables 2, 3, 6, 7)
  3. Five-group Kruskal-Wallis tests + Dunn-Bonferroni post hoc
(exploratory)
  4. Within-animal paired comparisons + exact signed-rank CIs (Table
4)
  5. Linear mixed model / ICC (Section 3.5)
  6. Body-weight correlations, tendon-level and animal-level (Section
3.1)
  7. Ultrasonographic thickness analyses incl. ANCOVA and animal-level
     sensitivity analysis (Section 3.9)
  8. Ex vivo measurements and Bland-Altman agreement (Section 3.10)
  9. Macroscopic findings: permutation test and Fisher test (Section
```

```
3.7)
 10. Agreement between modalities, incl. three-month-restricted
comparison
     (Section 3.11)
 11. Benjamini-Hochberg FDR correction across the 15 treatment-related
tests
     (Sections 2.18, 3.4, 3.7, 3.9)
 12. Post hoc sensitivity / minimum-detectable-effect analysis
(Section 2.4)
"""
import itertools
import numpy as np
from scipy import stats

#
---------------------------------------------------------------------
------
# 1. RAW DATA -- one row per tendon:
# (id, group, weight_kg, morphology, cellularity, vascularity,
fibrosis,
#  composite, US_day1_mm, US_day14_mm, US_3mo_mm|None, PM_US20_mm,
#  caliper20_mm, caliper35_mm, adhesions(0/1), hyperaemia(0/1),
#  US_abnormal_day14(0/1), US_abnormal_3mo(0/1), US_abnormal_any(0/1))
#
---------------------------------------------------------------------
------
D = [
("560DR","oil",4.23,0,0,2,2,4,6.2,5.4,None,4.1,5.0,9.2,1,0,0,0,0),
("563DR","oil",4.40,3,1,1,0,5,5.7,5.4,None,4.9,5.8,9.5,0,0,0,1,1),
("565DR","oil",3.93,1,1,0,0,2,6.0,5.4,None,4.2,4.0,8.1,1,0,0,0,0),
("566DR","oil",4.07,0,0,1,2,3,5.9,5.6,None,4.0,4.4,8.7,0,0,0,0,1),
("570DR","oil",4.74,0,0,0,1,1,6.1,7.2,None,4.9,6.9,8.0,1,0,0,1,1),
("574DR","oil",3.91,0,0,0,0,0,6.1,5.4,None,4.1,5.4,9.0,1,0,0,0,0),
("794DR","oil",3.98,0,0,1,1,2,5.5,5.6,None,4.9,5.0,9.5,1,0,0,0,0),
("551D","ctrl",4.05,1,0,2,1,4,6.3,5.9,None,4.4,4.4,8.6,1,0,0,1,1),
("554D","ctrl",4.12,3,2,1,0,6,6.2,5.7,None,4.1,6.1,9.5,0,1,0,1,1),
("559D","ctrl",3.40,0,1,1,0,2,6.0,5.8,None,3.9,4.4,8.5,0,1,0,1,1),
("572D","ctrl",4.00,1,1,2,1,5,6.0,5.8,None,4.3,4.4,8.9,0,1,1,0,1),
("777D","ctrl",4.09,0,0,0,0,0,6.6,6.7,None,4.7,5.3,8.9,0,0,0,0,0),
("788D","ctrl",4.34,1,0,2,0,3,5.4,6.1,None,3.8,4.5,6.8,1,0,0,0,0),
("795D","ctrl",3.89,0,0,2,0,2,6.6,5.7,None,3.4,5.1,10.3,0,0,0,0,0),
("552D","vite",4.19,2,2,2,2,8,6.2,7.0,None,3.8,3.7,6.0,0,0,0,0,0),
("552S","vite",4.19,1,1,0,0,2,5.3,6.9,None,3.7,3.5,5.3,0,0,0,0,0),
("553D","vite",3.68,1,3,3,1,8,5.7,5.7,None,4.2,4.4,6.3,0,0,0,0,0),
("553S","vite",3.68,0,0,0,0,0,5.9,6.4,5.8,3.3,4.4,6.1,0,0,1,0,1),
("556D","vite",3.94,0,0,0,0,0,5.3,4.8,None,3.5,4.1,6.6,0,0,0,0,0),
("556S","vite",3.94,1,2,1,0,4,4.2,4.9,None,3.7,4.9,6.0,1,0,1,0,1),
("567D","vite",4.30,0,0,0,1,1,5.0,6.6,None,3.7,3.9,6.0,0,0,1,0,1),
("567S","vite",4.30,3,0,0,2,5,4.6,5.3,None,4.0,3.7,6.0,0,0,0,0,0),
("573D","vite",4.50,1,1,1,2,5,5.4,7.4,None,4.8,3.9,5.6,0,0,0,0,0),
```

```
("573S","vite",4.50,0,0,0,0,0,4.4,7.0,None,3.7,3.3,5.0,0,0,1,0,1),
("779D","vite",4.35,1,3,0,2,6,5.7,5.5,None,3.0,4.3,7.2,1,0,1,0,1),
("779S","vite",4.35,2,1,2,2,7,5.1,5.3,None,3.5,4.4,6.0,0,0,0,1,1),
("784D","vite",4.19,0,0,1,0,1,4.5,5.4,6.0,2.6,3.9,6.8,0,0,0,0,0),
("784S","vite",4.19,1,1,1,0,3,5.1,5.2,5.1,3.0,4.0,6.3,0,0,0,0,0),
("551P","prp",4.05,2,2,1,1,6,6.0,5.0,None,4.5,5.4,8.6,1,0,0,0,1),
("554P","prp",4.12,0,0,0,2,2,6.4,5.6,None,4.7,5.0,7.8,0,1,0,1,1),
("559P","prp",3.40,0,0,1,0,1,5.7,4.0,None,3.7,4.8,8.3,0,0,0,0,1),
("572P","prp",4.00,0,0,0,2,2,6.1,5.1,None,4.0,4.8,9.0,0,0,0,0,0),
("777P","prp",4.09,2,3,2,1,8,5.6,5.8,None,4.2,5.0,8.4,0,0,0,0,0),
("788P","prp",4.34,3,2,0,2,7,5.8,6.1,None,4.3,4.9,9.4,0,0,0,1,1),
("795P","prp",3.89,0,1,3,0,4,6.0,5.4,None,4.8,5.8,9.3,0,0,0,0,0),
("560C","col",4.23,1,1,2,0,4,6.2,6.0,None,4.3,6.2,7.9,0,0,0,1,1),
("563C","col",4.40,2,2,2,2,8,6.1,6.0,None,4.7,5.3,11.2,0,0,0,0,0),
("565C","col",3.93,1,1,2,0,4,6.0,6.6,None,5.4,5.7,8.6,1,0,0,0,0),
("566C","col",4.07,3,3,1,0,7,6.3,6.3,None,4.3,5.0,7.8,1,0,0,0,1),
("570C","col",4.74,0,0,2,2,4,5.5,5.2,None,4.4,5.0,9.5,0,1,1,0,1),
("574C","col",3.91,0,0,0,1,1,9.5,10.2,None,4.6,4.8,8.7,1,0,1,1,1),
("794C","col",3.98,3,2,1,0,6,5.6,5.1,None,4.9,5.5,10.0,0,0,0,0,0),
]
GROUPS = ["oil", "ctrl", "vite", "prp", "col"]
NAMES = {"oil": "Essential oils + ASTM", "ctrl": "Levofloxacin-only
control",
         "vite": "Vitamin E + selenium", "prp": "PRP", "col":
"Injectable collagen"}
IDX = dict(w=2, mor=3, cel=4, vas=5, fib=6, tot=7, us1=8, us14=9,
us3=10,
           pm=11, c20=12, c35=13, adh=14, hyp=15, ab14=16, ab3=17,
abany=18)

def by(grp, key):
    return [r[IDX[key]] for r in D if r[1] == grp]

def allv(key):
    return [r[IDX[key]] for r in D]

def med_iqr(v):
    return (np.median(v), np.percentile(v, 25), np.percentile(v, 75))

print("=" * 78)
print("2. DESCRIPTIVE SUMMARIES")
print("=" * 78)
for grp in GROUPS:
    m, lo, hi = med_iqr(by(grp, "tot"))
    print(f"  {NAMES[grp]:28s} composite median {m:.2f} [{lo:.2f}-
{hi:.2f}] "
          f"mean {np.mean(by(grp,'tot')):.2f} +/-
{np.std(by(grp,'tot'),ddof=1):.2f}")
for dom in ("mor", "cel", "vas", "fib"):
    row = "  ".join(f"{med_iqr(by(g,dom))[0]:.2f}[{med_iqr(by(g,dom))
```

```
[1]:.2f}-{med_iqr(by(g,dom))[2]:.2f}]" for g in GROUPS)
    print(f"  domain {dom}: {row}")

print("=" * 78)
print("3. FIVE-GROUP KRUSKAL-WALLIS (exploratory; Section 3.2-3.3)")
print("=" * 78)
for key, label in [("tot", "composite"), ("mor", "morphology"),
("cel", "cellularity"),
                   ("vas", "vascularity"), ("fib", "fibrosis")]:
    H, p = stats.kruskal(*[by(g, key) for g in GROUPS])
    print(f"  {label:12s} H = {H:.3f}, p = {p:.2f}")
# epsilon-squared for the composite
H, p = stats.kruskal(*[by(g, "tot") for g in GROUPS])
n = len(D)
eps2 = max(0.0, (H - 4) / (n - 5))  # (H - k + 1)/(n - k)
print(f"  composite epsilon-squared ~= {eps2:.3f}")
# Dunn post hoc with Bonferroni (10 pairwise comparisons)
ranks = stats.rankdata(allv("tot"))
gr = [r[1] for r in D]
rbar = {g: np.mean([rk for rk, gg in zip(ranks, gr) if gg == g]) for g
in GROUPS}
ng = {g: gr.count(g) for g in GROUPS}
ties = np.unique(allv("tot"), return_counts=True)[1]
tie_corr = 1 - (np.sum(ties**3 - ties)) / (n**3 - n)
dunn = []
for a, b in itertools.combinations(GROUPS, 2):
    se = np.sqrt((n * (n + 1) / 12) * tie_corr * (1 / ng[a] + 1 /
ng[b]))
    z = abs(rbar[a] - rbar[b]) / se
    praw = 2 * (1 - stats.norm.cdf(z))
    dunn.append((a, b, min(1.0, praw * 10)))
best = min(dunn, key=lambda t: t[2])
print(f"  Dunn-Bonferroni: smallest adjusted p = {best[2]:.2f}
({NAMES[best[0]]} vs {NAMES[best[1]]})")

print("=" * 78)
print("4. WITHIN-ANIMAL PAIRED COMPARISONS (Section 3.4, Table 4)")
print("=" * 78)
def exact_signrank_ci(diffs, alpha=0.05):
    """Exact signed-rank confidence interval from Walsh averages (n <=
12)."""
    d = sorted(diffs); nn = len(d); M = nn * (nn + 1) // 2
    walsh = sorted((a + b) / 2 for a, b in
itertools.combinations_with_replacement(d, 2))
    counts = np.zeros(M + 1)
    for mask in range(2 ** nn):
        counts[sum((i + 1) for i in range(nn) if mask >> i & 1)] += 1
    counts /= counts.sum(); cum = np.cumsum(counts)
    k = 0
    while cum[k] <= alpha / 2:
```

```
        k += 1
    return walsh[k - 1], walsh[M - k], 1 - 2 * cum[k - 1]

tot = {r[0]: r[IDX["tot"]] for r in D}
pairs_co = [("560C","560DR"),("563C","563DR"),("565C","565DR"),
("566C","566DR"),
            ("570C","570DR"),("574C","574DR"),("794C","794DR")]
pairs_pc = [("551P","551D"),("554P","554D"),("559P","559D"),
("572P","572D"),
            ("777P","777D"),("788P","788D"),("795P","795D")]
for label, pp in [("collagen - essential oils", pairs_co), ("PRP -
levofloxacin-only control", pairs_pc)]:
    diffs = [tot[a] - tot[b] for a, b in pp]
    nz = [x for x in diffs if x != 0]
    w = stats.wilcoxon(nz, method="exact")
    lo, hi, cov = exact_signrank_ci(diffs)
    print(f"  {label}: diffs {diffs}, median {np.median(diffs):+.1f},
"
          f"exact p = {w.pvalue:.3f} (Bonferroni x2 = {min(1,
2*w.pvalue):.3f}), "
          f"exact {cov*100:.1f}% CI [{lo:+.1f}, {hi:+.1f}]")
# vitamin E left vs right symmetry
vl = {r[0][:3]: r[IDX["tot"]] for r in D if r[1] == "vite" and
r[0].endswith("S")}
vr = {r[0][:3]: r[IDX["tot"]] for r in D if r[1] == "vite" and
r[0].endswith("D")}
keys = sorted(vl)
dl = [vl[k] for k in keys]; dr = [vr[k] for k in keys]
w = stats.wilcoxon([a - b for a, b in zip(dl, dr) if a != b])
rho, prho = stats.spearmanr(dl, dr)
print(f"  vitamin E left vs right: medians {np.median(dl):.1f} vs
{np.median(dr):.1f}, "
      f"Wilcoxon p = {w.pvalue:.2f}, Spearman rho = {rho:.3f} (p =
{prho:.2f})")

print("=" * 78)
print("5. LINEAR MIXED MODEL / ICC (Section 3.5)")
print("=" * 78)
try:
    import statsmodels.formula.api as smf
    import pandas as pd
    df = pd.DataFrame({"tot": allv("tot"), "grp": gr, "animal": [r[0]
[:3] for r in D]})
    md = smf.mixedlm("tot ~ C(grp, Treatment('ctrl'))", df,
groups=df["animal"]).fit(reml=True)
    var_a = float(md.cov_re.iloc[0, 0]); var_e = float(md.scale)
    print(f"  between-animal variance = {var_a:.4f}, residual =
{var_e:.4f}, "
          f"ICC = {var_a/(var_a+var_e):.3f} (boundary estimate; see
Section 3.5)")
```

```
    for name, co in md.fe_params.items():
        if "grp" in name:
            print(f"  fixed effect {name}: {co:+.2f}")
except Exception as e:
    print("  statsmodels unavailable or model failed:", e)

print("=" * 78)
print("6. BODY WEIGHT (Section 3.1)")
print("=" * 78)
rho, p = stats.spearmanr(allv("w"), allv("tot"))
print(f"  tendon-level (n=42, weights duplicated): rho = {rho:.3f}, p
= {p:.2f}")
animals = {}
for r in D:
    animals.setdefault(r[0][:3], {"w": r[IDX['w']], "t": []})
["t"].append(r[IDX["tot"]])
aw = [v["w"] for v in animals.values()]; at = [np.mean(v["t"]) for v
in animals.values()]
rho, p = stats.spearmanr(aw, at)
print(f"  animal-level (n=21, mean of two tendons): rho = {rho:.3f}, p
= {p:.2f}")

print("=" * 78)
print("7. IN VIVO THICKNESS (Section 3.9)")
print("=" * 78)
H, p = stats.kruskal(*[by(g, "us1") for g in GROUPS])
print(f"  baseline thickness KW: p = {p:.3f}")
ch = {g: [round(a - b, 2) for a, b in zip(by(g, "us14"), by(g,
"us1"))] for g in GROUPS}
H, p = stats.kruskal(*[ch[g] for g in GROUPS])
print(f"  absolute change KW: p = {p:.3f}")
rel = {g: [100 * (a - b) / b for a, b in zip(by(g, "us14"), by(g,
"us1"))] for g in GROUPS}
H, p = stats.kruskal(*[rel[g] for g in GROUPS])
print(f"  relative change KW: p = {p:.3f}")
for g_ in GROUPS:
    nz = [x for x in ch[g_] if x != 0]
    w = stats.wilcoxon(nz) if nz else None
    print(f"    {NAMES[g_]:28s} median change {np.median(ch[g_]):+.2f}
mm, within-group p = "
          f"{w.pvalue:.3f}" if w else "    (all zero)")
rho, p = stats.spearmanr(allv("us1"), [a - b for a, b in
zip(allv('us14'), allv('us1'))])
print(f"  regression to the mean: rho = {rho:.2f}, p = {p:.3f}")
# ANCOVA: day14 ~ baseline + group  (partial F-test vs baseline-only
model)
y = np.array(allv("us14")); x = np.array(allv("us1"))
Gd = np.zeros((n, 4)); order_ = [g_ for g_ in GROUPS if g_ != "ctrl"]
for j, g_ in enumerate(order_):
    Gd[:, j] = [1 if r[1] == g_ else 0 for r in D]
```

```
X0 = np.column_stack([np.ones(n), x])
X1 = np.column_stack([X0, Gd])
b0, res0 = np.linalg.lstsq(X0, y, rcond=None)[:2]
b1, res1 = np.linalg.lstsq(X1, y, rcond=None)[:2]
rss0, rss1 = float(res0[0]), float(res1[0])
df1, df2 = 4, n - X1.shape[1]
F = ((rss0 - rss1) / df1) / (rss1 / df2)
pF = 1 - stats.f.cdf(F, df1, df2)
print(f"  ANCOVA baseline beta = {b1[1]:.2f}; group effect F({df1},
{df2}) = {F:.2f}, p = {pF:.3f}")
XtX_inv = np.linalg.inv(X1.T @ X1); s2 = rss1 / df2
j_vite = 2 + order_.index("vite")
se = np.sqrt(s2 * XtX_inv[j_vite, j_vite])
tstat = b1[j_vite] / se
print(f"  vitamin E contrast vs control: {b1[j_vite]:+.2f} mm, p =
{2*(1-stats.t.cdf(abs(tstat), df2)):.3f}")
# animal-level sensitivity analysis, vitamin E group
va = {}
for r in D:
    if r[1] == "vite":
        va.setdefault(r[0][:3], []).append(r[IDX["us14"]] -
r[IDX["us1"]])
chA = [round(np.mean(v), 3) for v in va.values()]
w = stats.wilcoxon([x for x in chA if x != 0], method="exact")
lo, hi, cov = exact_signrank_ci(chA)
print(f"  vitamin E animal-level (n=7): median {np.median(chA):+.2f}
mm, exact p = {w.pvalue:.3f}, "
      f"exact {cov*100:.1f}% CI [{lo:+.2f}, {hi:+.2f}]")
rho, p = stats.spearmanr([a - b for a, b in zip(allv('us14'),
allv('us1'))], allv("tot"))
print(f"  thickness change vs composite score: rho = {rho:.2f}, p =
{p:.2f}")

print("=" * 78)
print("8. EX VIVO MEASUREMENTS AND METHOD AGREEMENT (Section 3.10;
descriptive)")
print("=" * 78)
w = stats.wilcoxon([a - b for a, b in zip(allv("c35"), allv("c20"))])
print(f"  caliper 35 mm vs 20 mm: medians {np.median(allv('c35')):.2f}
vs "
      f"{np.median(allv('c20')):.2f} mm, Wilcoxon p = {w.pvalue:.2e}")
diffs = [a - b for a, b in zip(allv("pm"), allv("c20"))]
bias = np.mean(diffs); sd = np.std(diffs, ddof=1)
print(f"  Bland-Altman: bias = {bias:.2f} mm, LoA = [{bias-
1.96*sd:.2f}, {bias+1.96*sd:.2f}]")
rho, p = stats.spearmanr(allv("pm"), allv("c20"))
print(f"  PM US vs caliper correlation: rho = {rho:.2f}, p = {p:.2e}")
rho, p = stats.spearmanr(allv("us14"), allv("pm"))
print(f"  in vivo day-14 vs post-mortem US: rho = {rho:.2f}, p =
{p:.2f}")
```

```
print("=" * 78)
print("9. MACROSCOPIC FINDINGS (Section 3.7)")
print("=" * 78)
anyf = [1 if (r[IDX["adh"]] or r[IDX["hyp"]]) else 0 for r in D]
obs = np.array([[sum(a for a, r in zip(anyf, D) if r[1] == g_),
                 sum(1 for a, r in zip(anyf, D) if r[1] == g_ and a ==
0)] for g_ in GROUPS])
chi_obs = stats.chi2_contingency(obs, correction=False)[0]
rng_ = np.random.default_rng(2024)
labels = np.array(gr); vals = np.array(anyf)
count = 0; B = 20000
for _ in range(B):
    perm = rng_.permutation(vals)
    tab = np.array([[perm[labels == g_].sum(), (perm[labels == g_] ==
0).sum()] for g_ in GROUPS])
    if stats.chi2_contingency(tab, correction=False)[0] >= chi_obs -
1e-12:
        count += 1
print(f"  permutation test (chi2 statistic, B = {B}): p =
{count/B:.3f}")
OR_table = [[5, 2], [2, 12]]  # adhesions oil 5/7 vs vitE 2/14
OR, pf = stats.fisher_exact(OR_table)
print(f"  adhesions, oils+ASTM vs vitamin E: Fisher p = {pf:.3f}, OR =
{OR:.1f}")
loc = [1 if r[1] in ("oil", "prp", "col") else 0 for r in D]
tab = [[sum(a for a, l in zip(anyf, loc) if l), sum(1 for a, l in
zip(anyf, loc) if l and not a)],
       [sum(a for a, l in zip(anyf, loc) if not l), sum(1 for a, l in
zip(anyf, loc) if not l and not a)]]
print(f"  locally treated vs other, any finding: Fisher p =
{stats.fisher_exact(tab)[1]:.2f}")

print("=" * 78)
print("10. AGREEMENT BETWEEN MODALITIES (Section 3.11)")
print("=" * 78)
ab = [r[IDX["abany"]] for r in D]
g1 = [t for t, a in zip(allv("tot"), ab) if a]; g0 = [t for t, a in
zip(allv("tot"), ab) if not a]
print(f"  US abnormal (n={len(g1)}) vs normal: medians
{np.median(g1):.1f} vs {np.median(g0):.1f}, "
      f"MW p = {stats.mannwhitneyu(g1, g0).pvalue:.2f}")
g1 = [t for t, a in zip(allv("tot"), anyf) if a]; g0 = [t for t, a in
zip(allv("tot"), anyf) if not a]
print(f"  macroscopic abnormal vs normal: MW p =
{stats.mannwhitneyu(g1, g0).pvalue:.2f}")
adh_ = [r[IDX["adh"]] for r in D]
g1 = [f for f, a in zip(allv("fib"), adh_) if a]; g0 = [f for f, a in
zip(allv("fib"), adh_) if not a]
print(f"  adhesions vs paratendinous fibrosis grade: medians
```

```
{np.median(g1):.1f} vs {np.median(g0):.1f}, "
      f"MW p = {stats.mannwhitneyu(g1, g0).pvalue:.2f}")
ab3 = [r[IDX["ab3"]] for r in D]
g1 = [t for t, a in zip(allv("tot"), ab3) if a]; g0 = [t for t, a in
zip(allv("tot"), ab3) if not a]
print(f"  RESTRICTED to 3-month abnormalities (n={len(g1)}): medians
{np.median(g1):.1f} vs "
      f"{np.median(g0):.1f}, MW p = {stats.mannwhitneyu(g1,
g0).pvalue:.2f}")

print("=" * 78)
print("11. BENJAMINI-HOCHBERG FDR ACROSS THE 15 TREATMENT-RELATED
TESTS")
print("=" * 78)
tests = {"KW composite": 0.45, "KW morphology": 0.62, "KW
cellularity": 0.34,
         "KW vascularity": 0.27, "KW fibrosis": 0.47, "paired
collagen-oils": 0.031,
         "paired PRP-control": 0.63, "vitE left-right": 0.53,
"macroscopic permutation": 0.031,
         "KW abs thickness change": 0.003, "KW rel thickness change":
0.003,
         "vitE within-group thickness": 0.007, "ANCOVA group effect":
0.006,
         "ANCOVA vitE contrast": 0.017, "adhesion Fisher": 0.017}
names = list(tests); ps = np.array([tests[k] for k in names]); m =
len(ps)
order_i = np.argsort(ps); adj = np.empty(m); prev = 1.0
for rank in range(m - 1, -1, -1):
    i = order_i[rank]
    prev = min(prev, ps[i] * m / (rank + 1)); adj[i] = prev
for k, praw, padj in sorted(zip(names, ps, adj), key=lambda t: t[1]):
    print(f"  {k:32s} p = {praw:<6g} BH-adjusted = {padj:.3f}")

print("=" * 78)
print("12. SENSITIVITY / MINIMUM DETECTABLE EFFECT (Section 2.4)")
print("=" * 78)
try:
    from statsmodels.stats.power import FTestAnovaPower, TTestPower
    an = FTestAnovaPower(); tt = TTestPower()
    f80 = an.solve_power(effect_size=None, nobs=42, alpha=0.05,
power=0.8, k_groups=5)
    print(f"  five-group MDES at 80% power: f = {f80:.2f} (eta^2 =
{f80**2/(1+f80**2):.2f})")
    print(f"  power at f = 0.25: {an.solve_power(effect_size=0.25,
nobs=42, alpha=0.05, k_groups=5):.2f}")
    print(f"  power at f = 0.40: {an.solve_power(effect_size=0.40,
nobs=42, alpha=0.05, k_groups=5):.2f}")
    dz80 = tt.solve_power(effect_size=None, nobs=7, alpha=0.05,
power=0.8)
```

```
    print(f"  paired MDES (n = 7 pairs) at 80% power: dz =
{dz80:.2f}")
    print(f"  power at dz = 0.8: {tt.solve_power(effect_size=0.8,
nobs=7, alpha=0.05):.2f}")
except Exception as e:
    print("  statsmodels unavailable:", e)
print("\nDone. Compare printed values against the revised
manuscript.")
```
